# A widespread inversion polymorphism conserved among *Saccharomyces* species is caused by recurrent homogenization of a sporulation gene family

**DOI:** 10.1371/journal.pgen.1010525

**Published:** 2022-11-28

**Authors:** Letal I. Salzberg, Alexandre A. R. Martos, Lisa Lombardi, Lars S. Jermiin, Alfonso Blanco, Kevin P. Byrne, Kenneth H. Wolfe

**Affiliations:** 1 Conway Institute, University College Dublin, Dublin, Ireland; 2 School of Medicine, University College Dublin, Dublin, Ireland; 3 School of Biomolecular and Biomedical Science, University College Dublin, Dublin, Ireland; 4 School of Biology and Environmental Science, University College Dublin, Dublin, Ireland; 5 Earth Institute, University College Dublin, Dublin, Ireland; 6 Research School of Biology, Australian National University, Canberra, Australian Capital Territory, Australia; University of Rochester, UNITED STATES

## Abstract

*Saccharomyces* genomes are highly collinear and show relatively little structural variation, both within and between species of this yeast genus. We investigated the only common inversion polymorphism known in *S*. *cerevisiae*, which affects a 24-kb ‘flip/flop’ region containing 15 genes near the centromere of chromosome XIV. The region exists in two orientations, called reference (REF) and inverted (INV). Meiotic recombination in this region is suppressed in crosses between REF and INV orientation strains such as the BY x RM cross. We find that the inversion polymorphism is at least 17 million years old because it is conserved across the genus *Saccharomyces*. However, the REF and INV isomers are not ancient alleles but are continually being re-created by re-inversion of the region within each species. Inversion occurs due to continual homogenization of two almost identical 4-kb sequences that form an inverted repeat (IR) at the ends of the flip/flop region. The IR consists of two pairs of genes that are specifically and strongly expressed during the late stages of sporulation. We show that one of these gene pairs, *YNL018C/YNL034W*, codes for a protein that is essential for spore formation. *YNL018C* and *YNL034W* are the founder members of a gene family, Centroid, whose members in other Saccharomycetaceae species evolve fast, duplicate frequently, and are preferentially located close to centromeres. We tested the hypothesis that Centroid genes are a meiotic drive system, but found no support for this idea.

## Author summary

We show that a region of the genome of *Saccharomyces* yeast species has toggled repeatedly between two alternative orientations for millions of years. The orientation of this 24-kb ’flip/flop’ region, near the centromere of chromosome XIV, is polymorphic in *S*. *cerevisiae*, *S*. *paradoxus*, and probably all other species of the genus *Saccharomyces*. Both orientations occur in laboratory and wild strains of *S*. *cerevisiae*, and meiotic recombination in the flip/flop region is suppressed in crosses between strains having opposite orientations such as S288C and SK1. The changes of orientation are caused by recombination between two sequences that form an inverted repeat flanking the flip/flop region. The inverted repeat contains two genes with roles in sporulation, but it is unclear what evolutionary pressure maintains the inverted repeat structure and causes the genes to undergo continual homogenization.

## Introduction

In budding yeasts such as *Saccharomyces cerevisiae* the extent of structural variation of the genome within each species is low, although a few examples of strain-specific genomic rearrangements have been discovered by long-read sequencing [1,2]. Inversion polymorphisms are a type of structural variation in which a section of a chromosome occurs in opposite orientations in different alleles. They are balanced rearrangements, so the alleles (orientation isomers) do not differ in their content of DNA but only in how it is arranged on the chromosome. Inversion polymorphisms are rare in budding yeasts, and to our knowledge the one studied in this paper is the only known example in *Saccharomyces* of a large inversion for which both orientations occur at a substantial frequency in natural populations. In *S*. *cerevisiae*, inversions engineered in the laboratory have been found to have substantial effects on the transcriptome and on fitness, and in some cases are lethal [3].

In other eukaryotes, naturally occurring inversion polymorphisms lead to suppression of meiotic recombination in the inverted region, and these are sometimes associated with the evolutionary formation of supergenes (clusters of linked genes that together determine a phenotype [4]) or with meiotic drive (inheritance of one allele in preference to others at the same locus, instead of normal 50:50 Mendelian ratios [5]). In the human genome many inversion polymorphisms have been identified, including a large one of 1.5 Mb on chromosome 17 [6] and numerous smaller ones [7], and some of these are associated with propensity to genetic diseases [8,9]. Inversion toggling–recurrent re-inversion of a region flanked by inverted repeats–has occurred during the evolution of humans, apes, and other mammals [6,10,11]. In some yeast species such as *Ogataea polymorpha*, an inversion polymorphism controls mating type, and inversion of the *MAT* region during mating-type switching is a regulated process [12,13].

**Fig 1:**
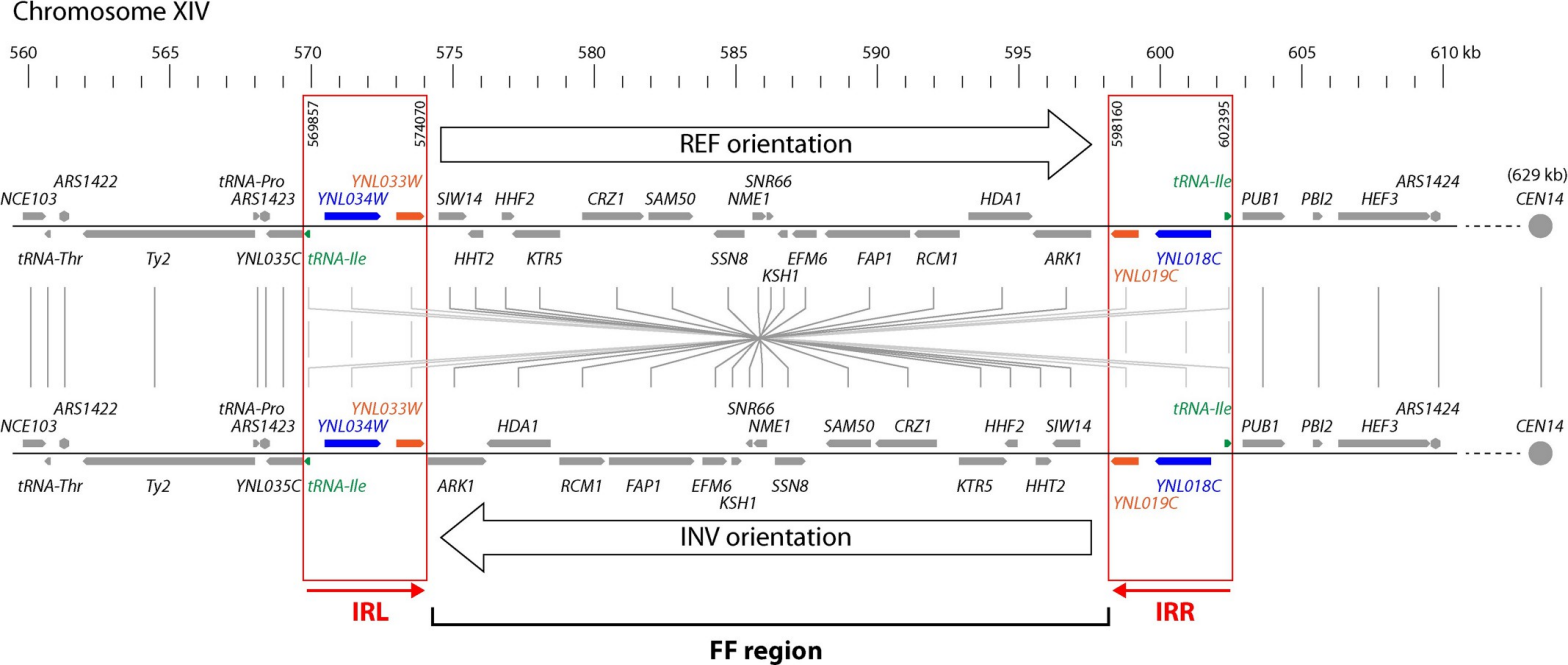
Organization of genes in the FF (flip/flop) region in REF and INV strains of *S*. *cerevisiae*. The IRL and IRR sequences form an inverted repeat, and recombination between them inverts the FF region. Coordinates correspond to the reference genome sequence of strain S288C, which has REF orientation.

When chromosome XIV of *Saccharomyces cerevisiae* was first sequenced, Philippsen *et al*. [14] noticed that it contained two almost identical copies of a 4.2-kb sequence, 24 kb apart and in opposite orientations. These inverted repeats (IRs) are of interest because they are the largest repeat sequence in the *S*. *cerevisiae* genome whose function is unknown (only the rDNA repeat, and repeats formed by Ty elements, are larger). We use the names IRL and IRR to refer to the left and right copies of the IR, respectively (Fig 1). Each copy begins at a tRNA gene (tRNA-Ile) and contains two protein-coding genes of unknown function: *YNL034W* and *YNL033W* in IRL, and *YNL019C* and *YNL018C* in IRR. In the *S*. *cerevisiae* reference genome (strain S288C), IRL and IRR have 97.6% nucleotide sequence identity, and this high level of identity extends throughout the intergenic regions as well as the coding regions. The protein pair Ynl034w/Ynl018c have 96% amino acid sequence identity, and the pair Ynl033w/Ynl019c have 99% identity, in the S288C reference sequence.

The 24-kb region between the two IRs occurs in opposite orientations in different strains of *S*. *cerevisiae* due to recombination between the IRs, so we refer to it as the FF (‘flip/flop’) region (Fig 1). We call the two orientations REF (reference, *i*.*e*. the same orientation as the *S*. *cerevisiae* S288C reference genome sequence), and INV (inverted relative to the reference). The FF region contains 15 genes, including two histone genes and two noncoding RNA genes (Table 1). Inversion of the FF region was first reported by Wei *et al*. [15] who sequenced the genome of the *S*. *cerevisiae* clinical isolate YJM789 and found that its FF region is inverted relative to the reference. Subsequently, other groups have noticed and commented on this inversion in other INV strains relative to the reference genome [1,16,17], but there has been no systematic analysis of its properties.

**Table 1:** Functions of genes located in the FF region and the IRs. Gray shading indicates genes located in the IRs; the other genes are in the FF region. Descriptions are based on *Saccharomyces* Genome Database annotations.

Systematic Name	Genetic Name	Function
*YNCN0015W*	*tI(AAU)N2*	tRNA-Ile
*YNL018C*	None	Unknown (Centroid family)
*YNL019C*	None	Unknown (Transmembrane protein)
*YNL020C*	*ARK1*	Ser/Thr kinase involved in regulation of cortical cytoskeleton and endocytosis
*YNL021W*	*HDA1*	Histone deacetylase, catalytic subunit
*YNL022C*	*RCM1*	rRNA m5C methyltransferase
*YNL023C*	*FAP1*	FKBP12-associated protein, confers rapamycin resistance by binding Fpr1
*YNL024C*	*EFM6*	Putative SAM-dependent lysine methyltransferase, methylates EF-1α
*YNL024C-A*	*KSH1*	“Kish” small membrane protein, role in secretory pathway
*YNCN0014W*	*SNR66*	snR66 small nucleolar RNA (snoRNA)
*YNCN0013W*	*NME1*	RNA component of RNAse MRP, cleaves pre-rRNA
*YNL025C*	*SSN8*	Cyclin-like component of RNA polymerase II holoenzyme
*YNL026W*	*SAM50*	Component of mitochondrial Sorting and Assembly Machinery complex
*YNL027W*	*CRZ1*	Transcription factor, activates stress response genes
*YNL029C*	*KTR5*	Putative mannosyltransferase involved in protein glycosylation
*YNL030W*	*HHF2*	Histone H4
*YNL031C*	*HHT2*	Histone H3
*YNL032W*	*SIW14*	Inositol phosphatase involved in inositol pyrophosphate metabolism
*YNL033W*	None	Unknown (Transmembrane protein)
*YNL034C*	None	Unknown (Centroid family)
*YNCN0012C*	*tI(AAU)N1*	tRNA-Ile

Here, we used a combination of data mining and new experiments to investigate the FF inversion polymorphism, with the aim of determining its prevalence, age, evolutionary dynamics, and phenotypic effects, and the reason for its maintenance. We find that the polymorphism is both ancient and recurrent. It is ancient because both orientations are present in multiple species of the genus *Saccharomyces*, so the region must have been polymorphic for at least 17 million years (the age of the oldest divergence between species in this genus [18]). However, it is also recurrent because the two orientation alleles were not formed by one ancient inversion event; instead, the FF region has changed orientation repeatedly, multiple times within each *Saccharomyces* species. Some diploid natural isolates are heterozygous for the two orientations. We show that the 4.2-kb IRs undergo continual homogenization, which causes the FF region to invert, but we were unable to determine why this homogenization occurs. We found that the two homogenized protein-coding genes located in the IR have roles in sporulation but are not involved in meiotic drive.

## Results

### The FF region exists in two orientations, REF and INV, in *Saccharomyces* species

**Fig 2:**
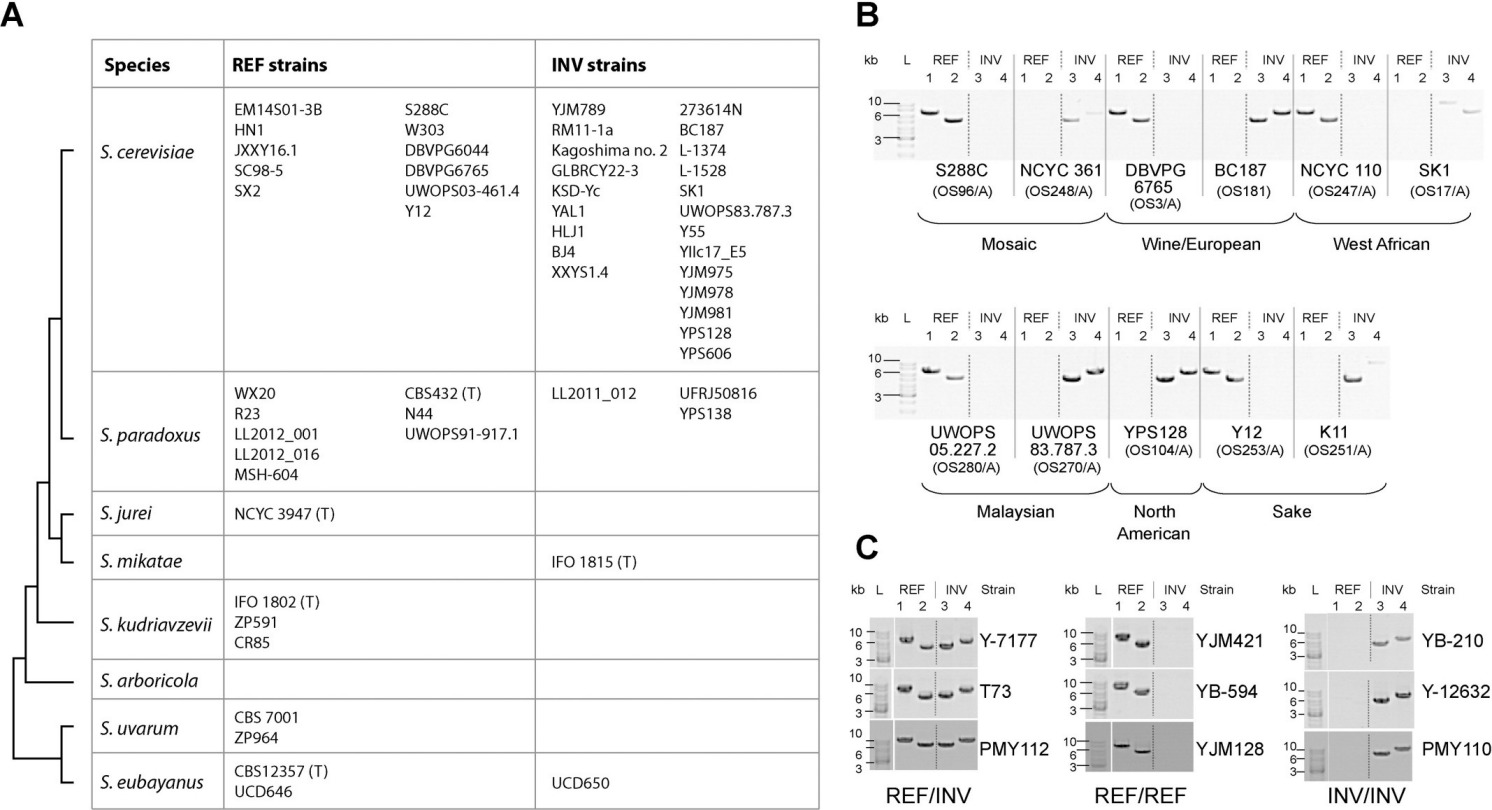
*Saccharomyces* species exhibit two orientations of the FF region, REF and INV. (A) Orientations of the FF region in long-read genome assemblies of strains of *Saccharomyces* species. The phylogram on the left indicates the species relationships. “(T)” denotes the type strain of the species. For *S*. *cerevisiae* and *S*. *paradoxus*, two columns are shown for each orientation: strains in the right column are strains used in the *Saccharomyces* Genome Resequencing Project (SGRP), and strains in the left column are not. References and accession numbers for long-read assemblies are given in S1 Table. (B) Examples of PCR assays of FF region orientation in SGRP strains of *S*. *cerevisiae*. For each strain, 4 PCRs were carried out using primers that bind in or near the genes on each side of IRR and IRL. The primer pairs are: 1, *YNL035C* + *SIW14*; 2, *ARK1* + *PUB1*; 3, *YNL035C* + *ARK1*; 4, *SIW14* + *PUB1*; a PCR product from PCRs 1 and 2 indicates REF orientation, and a PCR product from PCRs 3 and 4 indicates INV orientation (see S1 Fig and Materials and Methods). (C) Examples of PCR assays of FF region orientation in 9 diploid isolates of *S*. *cerevisiae* whose genomes have high nucleotide heterozygosity at SNP sites.

We analyzed the genomic organization around the FF region in the available genome sequences of strains of *S*. *cerevisiae* and other species of *Saccharomyces*, focusing only on assemblies in which chromosome XIV was assembled as a single contig. These data are mostly derived from long-read sequencing technologies (PacBio or Oxford Nanopore), usually in combination with short reads (e.g. Illumina) for error correction. In contrast, genome assemblies based on short-read technologies alone are usually not informative about the orientation of the FF region, because the length and high sequence identity of the IRs tends to cause assemblies to break at the junctions between the IRs and single-copy regions, making it impossible to determine the orientation. Short-read assemblies also tend to obscure any sequence differences between IRL and IRR because they usually merge the IRs into a single 4.2-kb IR contig whose coverage is twice the genome average. We examined all the available long-read assemblies for *S*. *cerevisiae* strains and found that 11 have REF orientation and 22 have INV orientation (Fig 2A). For one other strain, UWOPS87.2421, the published assembly [19] contains a gap and a partial duplication near the FF region, suggestive of misassembly. We used BLASTN to search the PacBio reads from which this assembly was generated, and found that reads with both REF and INV arrangements of the junctions between the IRs and single-copy regions are present, so we scored UWOPS87.2421 as a REF/INV heterozygote (S1 Table). Since this strain is thought to be haploid [19], it is possibly aneuploid for chromosome XIV.

We found that a similar pair of IRs is present at the same place in the genomes of all seven other species in the genus *Saccharomyces* (see Fig 2A for a phylogenetic tree). Within each species, the two copies of the IR are almost identical to each other, whereas there is extensive sequence divergence between species (e.g., 77% DNA sequence identity between the IRs of *S*. *cerevisiae* and *S*. *paradoxus*, which is similar to the genome average, 86% [20]). For *S*. *paradoxus*, both orientations of the FF region are present in the available long-read assemblies of different strains (8 REF strains, 3 INV strains; Fig 2A). For *S*. *eubayanus*, the long-read assembly of CBS12357, which is the type strain of this species (*i*.*e*., the strain on which the definition of the species is based), has REF orientation. We recently isolated two new isolates of *S*. *eubayanus* from Irish soil samples [21] and sequenced their genomes using Oxford Nanopore MinION long reads followed by short-read error correction. One of these isolates is REF and the other is INV (UCD646 and UCD650, respectively; Fig 2A), even though the two isolates differ by only 2,517 homozygous SNPs and came from soil samples located only 17 meters apart [21]. Long-read assemblies are available for only a few strains of other *Saccharomyces* species, but the type strain of *S*. *mikatae* has INV orientation whereas the available assemblies of *S*. *jurei*, *S*. *kudriavzevii*, and *S*. *uvarum* strains are all REF (Fig 2A).

To estimate the frequencies of the two orientations in populations of *S*. *cerevisiae* and *S*. *paradoxus*, we used PCR assays to determine FF region orientation in 36 *S*. *cerevisiae* strains and 23 *S*. *paradoxus* strains from the *Saccharomyces* Genome Resequencing Project (SGRP [22]) (S1 Table). PCR assays are shown in Figs 2B and S1. Long read assemblies became available for some of the SGRP strains after we had assayed them by PCR, and we found no discrepancies between the results from the two methods (S1 Table). Among the *S*. *cerevisiae* SGRP strains, 14 (39%) had REF orientation, 18 (50%) had INV orientation, and 4 (11%) gave both types of PCR product and so were scored as REF/INV heterozygotes (S1 Table). Among the *S*. *paradoxus* SGRP strains, 8 (35%) were REF and 15 (65%) were INV (S1 Table and S1 Fig). We also analyzed data from 24 natural isolates of *S*. *cerevisiae* from Taiwan sequenced by Nanopore [23], and found that 7 (29%) had REF orientation and 17 (71%) had INV orientation (S2 Table).

We also used PCR to assay 8 natural isolates of *S*. *uvarum* [24], of which 7 (87%) were REF and 1 (13%) was INV (S1 Table and S1 Fig). Thus, the FF region’s orientation is polymorphic in at least four species (*S*. *cerevisiae*, *S*. *paradoxus*, *S*. *eubayanus* and *S*. *uvarum*), and we suggest that it is likely to be polymorphic in all eight species of the genus *Saccharomyces* because IRs are present in every species.

The sequencing and PCR assays described above were carried out on haploid or highly homozygous diploid strains. To investigate whether REF/INV heterozygotes are found in diploid natural isolates, we examined some diploid *S*. *cerevisiae* isolates for which short-read genome sequencing or RAD-seq data indicated that they have significant heterozygosity at SNP positions and therefore must have been formed by outcrossing [25,26]. By PCR assays we found that some of these isolates are REF/INV heterozygotes, whereas others are REF/REF or INV/INV homozygotes (Figs 2C and S1). We also found that some diploid isolates with relatively low levels of SNP heterozygosity are REF/INV heterozygotes (for example, strain NRRL YB-908; S1 Fig).

We compared gene order (synteny) relationships around the FF region between *Saccharomyces* and other species, including species that diverged before, or after, the Whole Genome Duplication (WGD) event that occurred in the lineage leading to *S*. *cerevisiae* [27]. Although the two protein-coding genes in the IR (*YNL018C/YNL034W* and *YNL019C/YNL033W*) do not have orthologs in non-*Saccharomyces* species, we find that there is a small IR (74 bp), formed by two tRNA-Ile genes in opposite orientations, at the ends of the FF-like region in non-WGD species, and also at the ends of the region of *S*. *cerevisiae* chromosome IX that is ‘sister’ to the FF region as a result of the WGD (S2 Fig). Therefore the IR seems to have expanded in *Saccharomyces*, by adding copies of the two protein-coding genes into a pre-existing IR formed by tRNA-Ile genes. Furthermore, the order of genes in the non-WGD species and in the paralogous chromosome IX region, as well as in the post-WGD species *Candida castellii*, all suggest that the *Saccharomyces* FF region originally had INV orientation before the IRs expanded and flip/flopping began (S2 Fig).

### Meiotic recombination in the FF region is suppressed in REF/INV heterozygotes

We expect that during meiosis in diploids that are REF/INV heterozygotes, a single crossover within the FF region would result in the formation of derivatives of chromosome XIV with gross defects that would make gametes inviable [28]. A single crossover would produce two isochromosomes (S3 Fig). One is a hairpin with two copies of the left part of the chromosome (everything from the left telomere to the FF region), with no centromere. The other is a hairpin with two copies of the right part of the chromosome (everything from the FF region to the right telomere), including two centromeres. Therefore we expect the observed level of meiotic recombination in the FF region to be greatly reduced in REF x INV crosses, relative to REF x REF and INV x INV crosses. The only viable crossovers expected in the FF region in REF x INV crosses are double crossovers, which should be rare.

**Fig 3:**
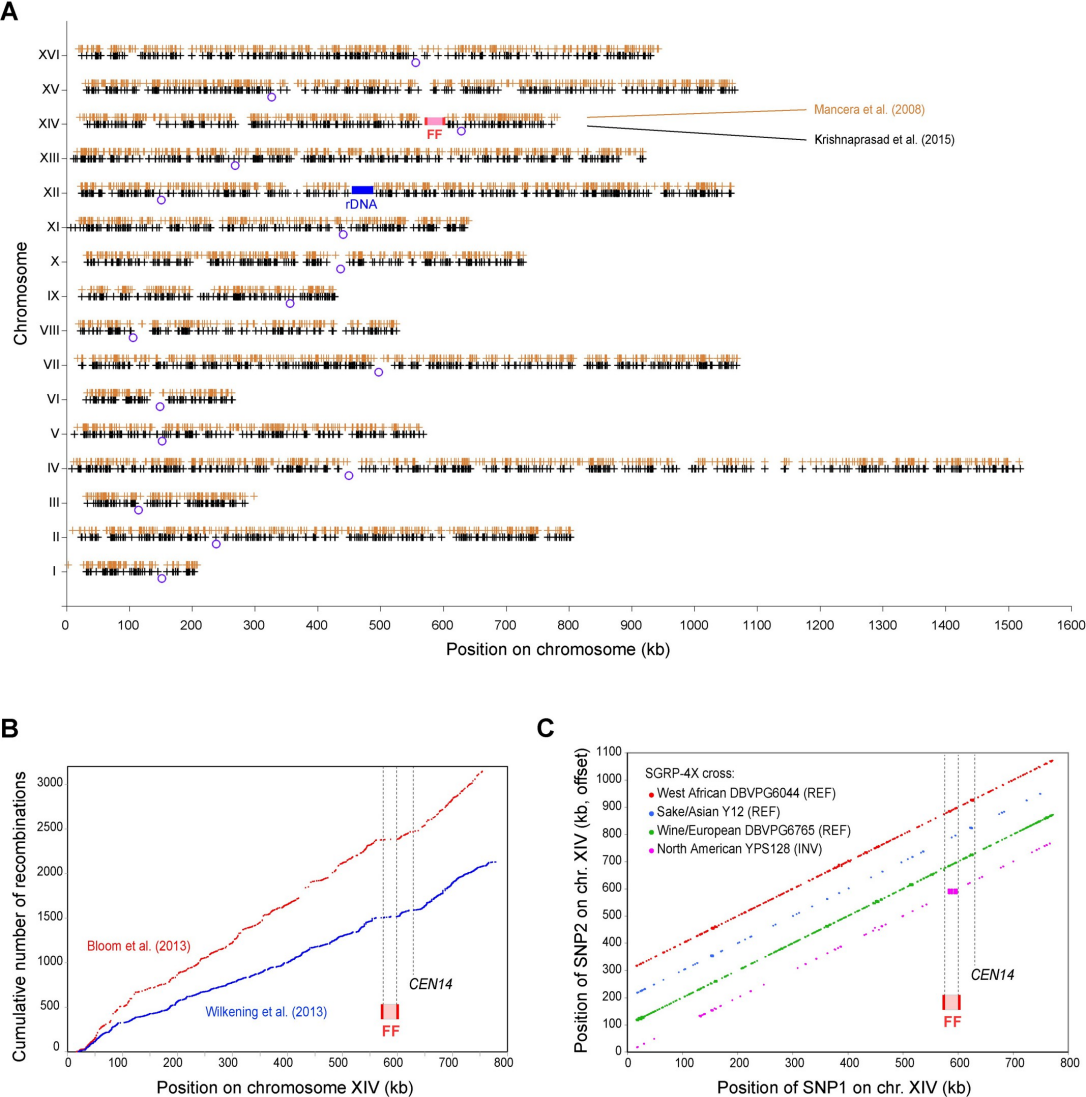
Meiotic recombination in the FF region is suppressed in *S*. *cerevisiae* REF x INV crosses. (A) Locations of meiotic crossovers on all chromosomes, in reanalysis of data from two S96 (REF) x YJM789 (INV) crosses. Orange crosses show the 3,235 crossovers in 50 complete tetrads from Mancera *et al*. [29], and black crosses show the 4,193 crossovers in 80 complete tetrads from Krishnaprasad *et al*. [30]. The FF (flip/flop) region on chromosome XIV is indicated in pink with the two IRs in red. Blue circles show centromere locations, and the blue rectangle marks the rDNA locus. (B) Recombination on chromosome XIV in random segregants from two REF x INV crosses: 1,008 segregants from a BY (REF) x RM (INV) cross [33] (red), and 720 segregants from an S96 (REF) x SK1 (INV) cross [31,32] (blue). The Y-axis shows the cumulative numbers of recombination events observed along chromosome XIV in each study. The FF region is seen to be a recombination coldspot. All the recombination events within it were investigated and found to be double crossovers. *CEN14* is also a coldspot, like all centromeres [29]. Gaps in the lines are caused by relatively long intervals between consecutive SNP markers, in which multiple spores can show recombination and cause the cumulative total to jump. (C) Linkage disequilibrium on chromosome XIV in the SGRP-4X cross. ‘Private’ SNP alleles in each of the four parental strains used in the SGRP-4X cross were identified (*i*.*e*., variants that are present in one parent but absent in the other three, so they can be used as tags to identify the parental source of DNA in a segregant). Points on each of the four diagonal lines show the locations of all pairs of private SNPs (SNP1 and SNP2) from a parent that remained in complete linkage disequilibrium after 12 generations of random mating (*i*.*e*., the pattern of presence/absence of the private allele from that parent at the SNP1 site was identical in all 175 F_12_ segregants to the pattern at the SNP2 site). The Y-axis is offset by 100 kb for different parents to make each diagonal visible. In most regions of the genome, only SNPs that are located very close to each other on the chromosome show such complete linkage, forming a diagonal. However, in the FF region, all the alleles private to the only INV strain in the cross (YPS128) remained in linkage disequilibrium because they did not recombine with the other strains, forming a block of co-segregating sites visible as a purple rectangle. In contrast, alleles private to each of the three REF parents in the FF region do not form blocks of co-segregating sites because these strains recombined with each other.

To investigate meiotic recombination in the FF region, we reanalyzed data from five published genetic cross experiments and found that each of them confirms that crossing over in the FF region is suppressed in REF x INV crosses. (*i*) The classic analysis of the recombination landscape in *S*. *cerevisiae* by Mancera *et al*. [29] used microarrays to map crossovers in 50 tetrads from a cross between the S288C derivative S96 (REF) and YJM789 (INV). (*ii*) Krishnaprasad *et al*. [30] used whole-genome sequencing to map crossovers in a further 80 tetrads from a cross between the same two parents. Plotting the locations of all 7,428 crossovers seen in these two experiments shows that there are only two large regions in the genome where crossovers did not occur: the rDNA locus on chromosome XII, and the FF region on chromosome XIV (Fig 3A).

Similarly, in random spore analyses, (*iii*) Wilkening *et al*. [31,32] sequenced 720 segregants from an S96 (REF) x SK1 (INV) cross, and (*iv*) Bloom *et al*. [33] sequenced 1,008 segregants from a BY (REF) x RM (INV) cross. These segregants again show an absence of meiotic recombination sites in the FF region (Fig 3B), except for a few sites that occurred in pairs and represent closely spaced double crossovers occurring within the FF region.

Additionally, (*v*) the SGRP-4X project was a four-way intercross among four parental strains representing pure geographic lineages of *S*. *cerevisiae* [34,35]. Three of the parental strains in this cross have REF orientation but the fourth (the North American isolate YPS128) has INV orientation. The genomes of 175 randomly chosen segregants were sequenced after 12 generations of random mating and meiosis [34,35]. Our analysis of the genotypes of the segregants shows that, in the FF region, the three REF strains recombined with each other during the 12 cycles of meiosis, but not with the INV strain (Fig 3C). SNPs in the FF region of the INV strain form a block of complete linkage disequilibrium in this cross because they were unable to recombine with the REF strains, whereas SNPs in the FF region of each REF strain do not form a block because they recombined with the other REF strains. From these five sets of results we conclude that meiotic recombination in the FF region of REF/INV heterozygotes is rare because single crossovers are likely to lead to derivatives of chromosome XIV that are inviable.

The BY x RM cross [36] and the YJM789 x S96 cross [37] have been used extensively to map quantitative trait loci (QTLs) or expression QTLs (eQTLs) in *S*. *cerevisiae*, so it is significant that they are REF x INV crosses. However, despite the large number of studies that have utilized these crosses [38–40], we do not know of any QTLs or eQTLs, for any trait, that have been mapped to sites in or near the FF region. The well-known chromosome XIV QTL hotspot centered on the pleiotropic gene *MKT1* [39,41,42] is located 100 kb to the left of the FF region. Therefore, the orientation of the FF region does not appear to cause any phenotypic difference between the REF and INV segregants from the BY x RM or YJM789 x S96 crosses, for any of the phenotypes that have been assayed in those studies, although it remains possible that it affects other unstudied phenotypes.

### SNP trees show multiple changes of FF orientation during the diversification of *S*. *cerevisiae* and *S*. *paradoxus* strains

How old is the FF inversion polymorphism? We have shown that the FF region exists in both orientations in at least 4 of the 8 species in the genus *Saccharomyces*, but this observation does not tell us how many times the region has become inverted during evolution. We considered three possible hypotheses about the polymorphism’s age. First, there might have been only one inversion event, in the common ancestor of all the *Saccharomyces* species. Second, there might have been separate inversion events in each species, but only one event per species. Third, the FF region might have undergone multiple inversions and re-inversions within each species, frequently changing from REF to INV orientation and back again.

**Fig 4:**
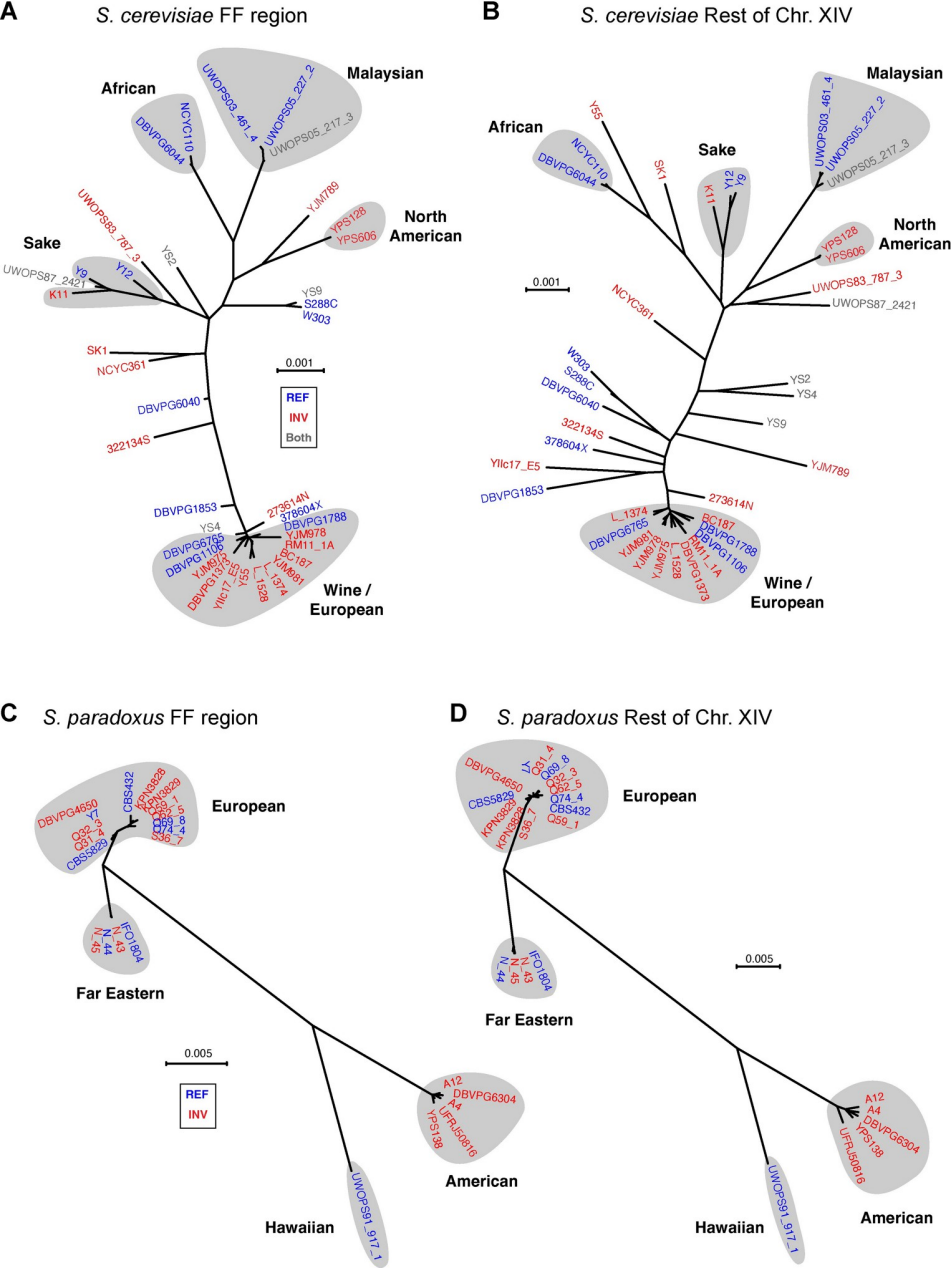
Phylogenetic analysis of parts of chromosome XIV shows that inversions of the FF region have occurred multiple times in *S*. *cerevisiae* and *S*. *paradoxus*. (A) *S*. *cerevisiae* tree constructed from the FF region. (B) *S*. *cerevisiae* tree constructed from the rest of chromosome XIV excluding the FF region and the IRs. (C) *S*. *paradoxus* tree constructed from the FF region. (D) *S*. *paradoxus* tree constructed from the rest of chromosome XIV excluding the FF region and the IRs. Strain names are shown in blue for REF orientations, red for INV orientations, and gray for strains that produced both REF and INV products in PCR assays. Phylogenetic trees were constructed as described in Materials and Methods.

To distinguish between these hypotheses, we analyzed genome sequence data of 38 *S*. *cerevisiae* strains whose FF region orientations are known, most of which come from the SGRP study [22]. We constructed two unrooted phylogenetic trees: one from the sequence of the 24-kb FF region (Fig 4A), and one from the sequence of the rest of chromosome XIV excluding the FF region and the IRs (Fig 4B). If the first or second hypothesis were correct, the unrooted tree inferred from the FF region should contain two clades–a REF clade and an INV clade–with the single inversion event occurring on the branch connecting these clades. However, this is not what is seen. Instead there are multiple REF and INV clades. The topology of the tree drawn from the FF region sequences agrees well with the tree drawn from the rest of chromosome XIV (Fig 4A and 4B). The FF region tree is also in good agreement with trees drawn from the whole genome sequences of these strains, and the major geographical lineages of *S*. *cerevisiae* are resolved in it [22]. The intermixing of REF and INV strains in Fig 4A indicates that multiple separate events of inversion of the FF region must have occurred during the evolution of the species *S*. *cerevisiae*, consistent with the third hypothesis. Two geographical populations of *S*. *cerevisiae* contain both REF and INV strains: the Wine/European clade, and the Sake clade (Fig 4A).

A similar analysis of 23 *S*. *paradoxus* strains leads to a similar conclusion for that species (Fig 4C and 4D). The phylogeny of its FF region agrees with the phylogeny of the rest of chromosome XIV, and the REF and INV orientations do not resolve into separate clades. Two geographical populations of *S*. *paradoxus* contain both REF and INV strains: the European clade, and the Far Eastern clade (Fig 4C).

It is clear from these phylogenetic trees that multiple inversions of the FF region have occurred during the diversification of both *S*. *cerevisiae* and *S*. *paradoxus*. It is not possible to count the number of events accurately due to the low resolution of the trees within the European populations of each species, but the distribution of orientations in the major lineages cannot be explained without a minimum of four inversion events in *S*. *cerevisiae* and three in *S*. *paradoxus*.

The FF region only rarely undergoes meiotic recombination in REF x INV crosses (Fig 3). If this leads to a reduced level of recombination in the FF region in natural populations, the region’s nucleotide diversity would be expected to be lower than in the rest of the genome due to the effect of background selection [43]. However, in Fig 4 the tree drawn from the FF region is approximately the same size as the tree drawn from the rest of chromosome XIV, both for *S*. *cerevisiae* (Fig 4A and 4B) and for *S*. *paradoxus* (Fig 4C and 4D), which suggests that (within each species) nucleotide diversity in the two parts of the chromosome is similar. We confirmed this observation by calculating π, the average nucleotide diversity among pairs of sequences in each set [44]. For *S*. *cerevisiae*, π = 0.00383 ± 0.00197 (mean ± s.d.) in the FF region and 0.00420 ± 0.00168 in the rest of chromosome XIV. For *S*. *paradoxus*, π = 0.01642 ± 0.01530 in the FF region and 0.01890 ± 0.01615 in the rest of the chromosome. Thus diversity is only marginally lower in the FF region in both species, perhaps indicating that REF x REF and INV x INV crosses are more frequent than REF x INV crosses in natural populations.

### Orientation of the FF region is stable on laboratory timescales

Even though the trees in Fig 4 show evidence that the FF region has changed orientation several times within each species, the pattern is not completely random and closely related strains tend to have the same orientation. We also examined data from some strains of *S*. *cerevisiae* that have been sequenced two or three times independently by different laboratories using long-read methods (the REF strains S288C [1,2] and W303 [45–47], and the INV strain Y55 [17,19]), and found no cases where the FF region’s orientation was different between two laboratories. These observations suggest that the orientation is relatively stable on shorter (non-evolutionary) timescales.

To investigate its stability in laboratory conditions, we made use of strains from a mutation accumulation experiment carried out by Nishant *et al*. [48], generously provided by Prof. Eric Alani. In this experiment, a diploid progenitor strain (SK1 genetic background, INV orientation) was used to initiate two types of mutation accumulation lines, vegetative and meiotic. Each vegetative line was propagated asexually through approximately 1,740 mitoses, and each meiotic line was propagated sexually through 50 meioses and approximately 1,000 mitoses [48]. We used PCR assays to determine the orientation of the FF region in the progenitor strain and the final strains from 20 vegetative lines and 19 meiotic lines, and found that they all have INV orientation (S4 Fig). Therefore we saw no change of orientation in a total of approximately 53,800 mitoses and 950 meioses, which indicates that the FF region’s orientation is stable under normal laboratory conditions.

### The Inverted Repeats flanking the FF region are homogenized within strains and species

**Fig 5:**
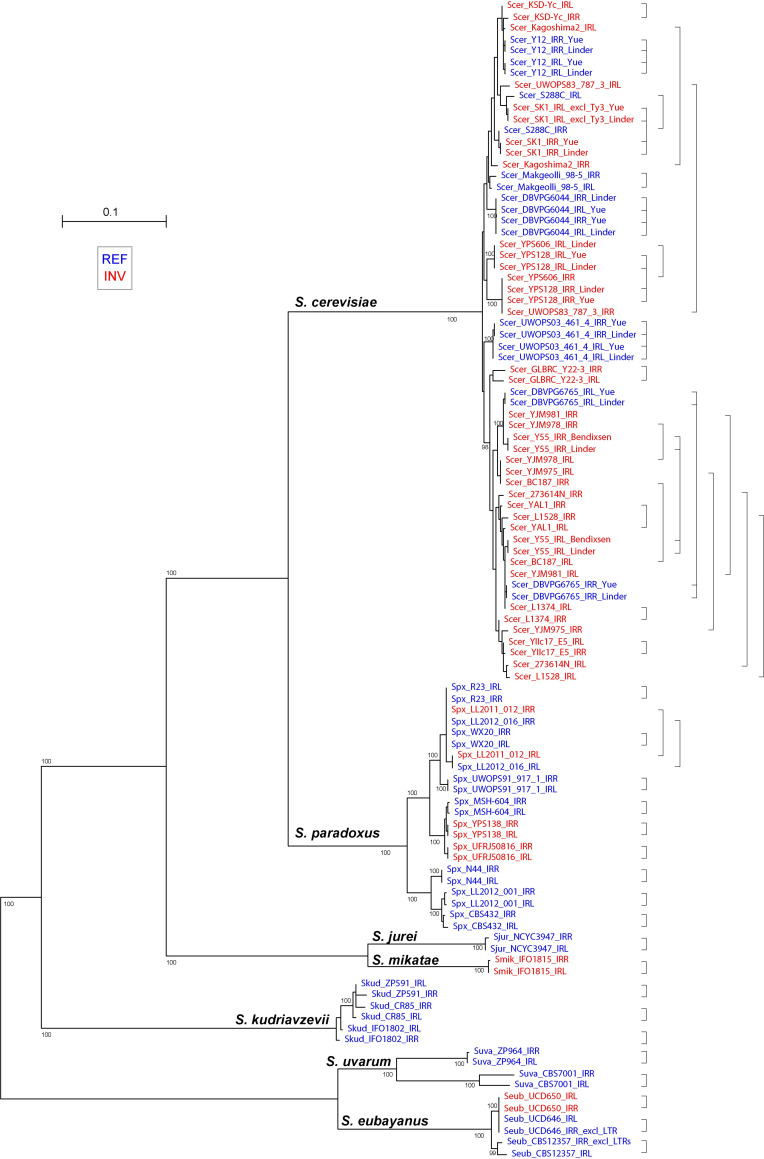
Phylogenetic tree of IR sequences from *Saccharomyces* strains that were sequenced using long-read technologies. Bootstrap support for key branches is shown. Strains named in blue have REF orientation, and strains named in red have INV orientation. Pairs of IRL and IRR sequences from the same strain are connected by the brace symbols on the right. For strains that were sequenced or assembled independently by two laboratories, the brace symbol connects four sequences, and author name is indicated for each sequence [1,17,19]. We use the names IRL and IRR to denote the left and right copy of the IR in each genome, regardless of whether the FF region is in REF or INV orientation. Thus IRR always refers to the copy of the IR that lies closer to the centromere, and IRL always refers to the more distal copy.

We constructed a phylogenetic tree from the DNA sequences of the two IR regions from each strain (IRL and IRR; Fig 1), using only genomes assembled by long-read sequencing. For almost all strains, the tree shows the IRL and IRR sequences clustering quite closely together, and in many cases they are each other’s closest relatives (Fig 5). This pattern indicates that the IRs have been homogenized frequently and are undergoing concerted evolution [49], so that high sequence identity of the IRs has been maintained within each strain whereas divergence has occurred among strains and among species. We considered the possibility that errors during the process of genome assembly might artifactually increase the sequence similarity between IRL and IRR within a strain, leading to apparent homogenization. However, for seven *S*. *cerevisiae* strains we were able to compare the IR sequences in two long-read assemblies that were generated independently by two different laboratories using different assembly methods [1,17,19]. In each case the data from the two laboratories was in agreement, even for strains such as *S*. *cerevisiae* DBVPG6765 in which IRL and IRR are not identical in sequence (Fig 5). From this result we conclude that the IR sequences in long read assemblies are generally accurate, and that IRL and IRR are continually being homogenized within strains and within species.

The phylogeny of the IR regions from different *Saccharomyces* species (Fig 5) has a topology matching the expected relationships among the species (Fig 2A; [18,50]). Within each species, strains with REF and INV orientations do not form separate clades in this IR tree, which is consistent with the conclusion from analysis of FF region sequences (Fig 4) that multiple changes of orientation have occurred within each species.

In three strains, the similarity between IRL and IRR is interrupted by the presence of a mobile genetic element in one of them, close to the 5’ end of the tRNA-Ile gene. There is a full-length Ty3 element 15 bp upstream of tRNA-Ile in IRL of *S*. *cerevisiae* strain SK1 [51], a solo Ty LTR 117 bp upstream of tRNA-Ile in IRR of *S*. *eubayanus* UCD646, and two consecutive Ty LTRs 40 bp upstream of tRNA-Ile in IRR of *S*. *eubayanus* CBS12357.

### The IR contains two sporulation-specific genes

We suggest that the recurring changes of orientation of the FF region are simply a by-product of the continual homogenization of the IRs by gene conversion, which occasionally causes crossing over and hence inversion of the FF region (see Discussion). We do not propose that the REF/INV polymorphism is being maintained by balancing selection to retain both of the orientations in yeast populations, but instead that natural selection is acting to maintain identical sequences in the two copies of the IR. Therefore we focused on the functions of the genes located in the IR, to try to understand why they are being homogenized. Each copy of the IR includes the complete coding sequences and promoters of two protein-coding genes–*YNL018C* and *YNL019C* in IRR, and *YNL033W* and *YNL034W* in IRL–as well as a tRNA-Ile gene on the opposite DNA strand (Fig 1). In the reference *S*. *cerevisiae* S288C genome the IR starts 10 bp downstream of the tRNA gene and ends 217 bp downstream of the stop codon of *YNL019C/YNL033W*, and its organization is almost identical in other strains and species.

There is little information about the functions of the protein-coding genes, but expression of both *YNL018C/YN034W* and *YNL019C/YNL033W* is sporulation-specific. Since the DNA sequences of *YNL034W* and *YNL018C* are almost identical (97.9% identity in *S*. *cerevisiae* S288C), and the sequences of *YNL033W* and *YNL019C* are almost identical (99.9%), the transcription levels of the individual genes in each pair cannot be determined using the available data from RNAseq or microarray experiments, so we cannot tell if there are transcriptional differences between IRL and IRR. Transcription of both *YNL018C/YN034W* and *YNL019C/YNL033W* was reported to be induced strongly in the middle stage of meiosis, continuing into sporulation, in several early microarray studies [52–54], and we confirmed that this profile is also seen in more recent meiotic RNAseq timecourse experiments [55,56] (S5A and S5B Fig). In contrast, in vegetative growth conditions most transcription of the IR region is antisense to the two protein-coding genes [56–58] (S5C Fig).

Lam *et al*. [59] found by microscopy of GFP fusion constructs that the protein products of both *YNL018C* and *YNL019C* are associated with the prospore membrane in *S*. *cerevisiae* (*YNL034W* and *YNL033W* were not tested). The two larger proteins encoded in the IRs, Ynl018c and Ynl034w, are both 612 amino acids long, with no protein domains predicted bioinformatically. The Ynl018c protein localizes to the peripheral region of the prospore membrane [59]. The two smaller proteins, Ynl019c and Ynl033w, are both 284 amino acids long and each of them contains two predicted transmembrane regions. Ynl019c protein was localized to the prospore membrane [59] and was predicted to be integral to the membrane because of its transmembrane domains. There is a third member of this transmembrane family in *S*. *cerevisiae*, Ypr027w, which is a 277 amino acid protein with 19% identity to Ynl019c/Ynl033w. Transcription of *YPR027W* is also induced during sporulation and the protein localizes to the prospore membrane [54,55,59]. Haploid strains with single knockouts of each of the four IR genes, or with double knockouts of the pairs of similar genes (*ynl018cΔ ynl034wΔ* or *ynl019cΔ ynl033wΔ* genotypes) showed no mutant phenotypes during vegetative growth [60].

### *YNL018C* and *YNL034W* are founder members of Centroid, a centromere-linked gene family in Saccharomycetaceae species

**Fig 6:**
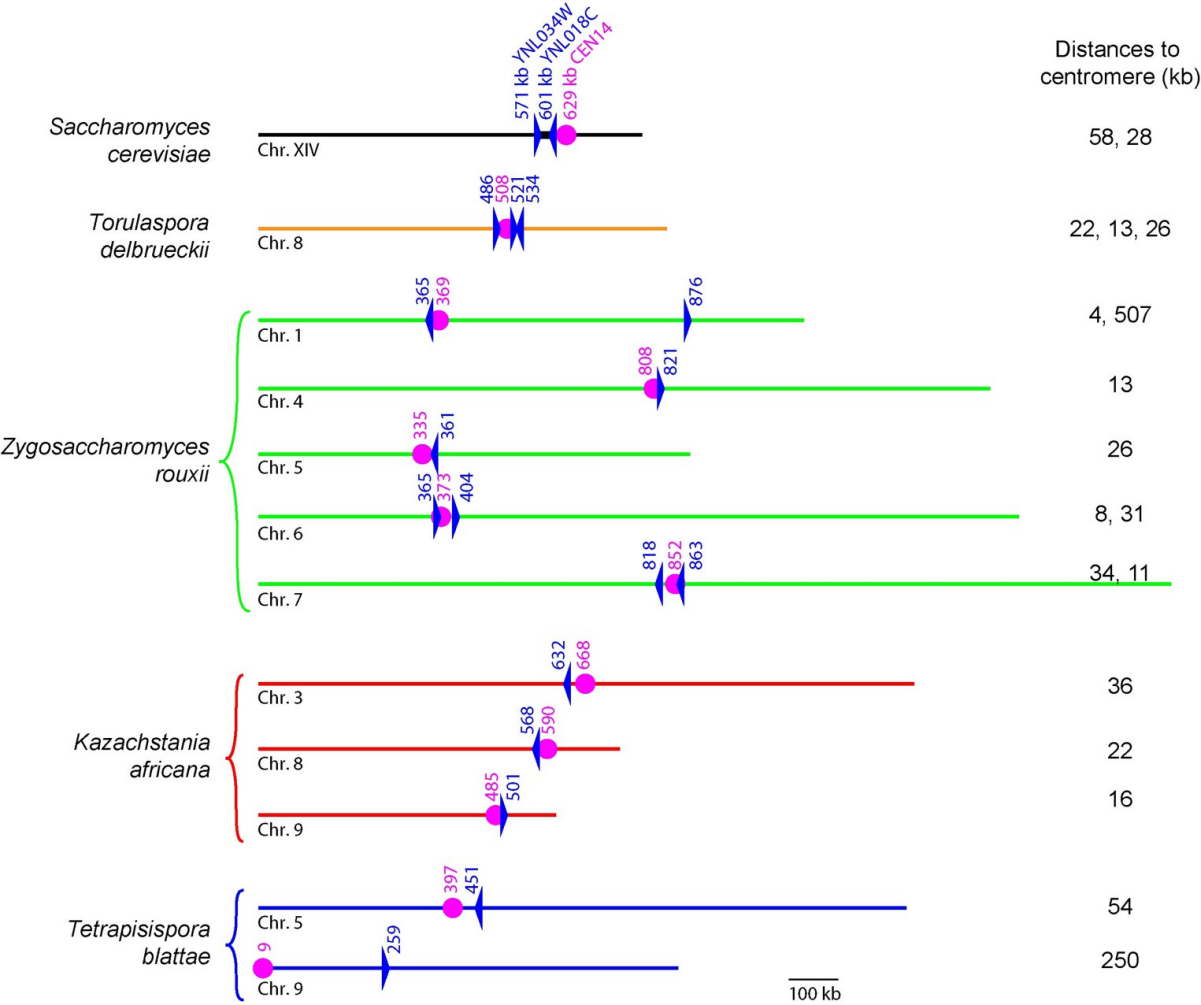
Locations of Centroid genes (homologs of *YNL018C* and *YNL034W*) relative to centromeres, on budding yeast chromosomes. Blue triangles show the location and orientation of Centroid genes, pink circles represent centromeres, and numbers above these symbols indicate their position (kb).

*YNL018C* and *YNL034W* have orthologs at equivalent genomic locations in every species of *Saccharomyces*, but not outside this genus (S2 Fig). By BLASTP searches we found non-syntenic homologs of these genes in a few other yeast species within the family Saccharomycetaceae, but none in more distantly related organisms. The homologs comprise a highly divergent gene family with multiple members in some species–for example, there are eight members of this family in the genome of *Zygosaccharomyces rouxii*, three in *Torulaspora delbrueckii*, and three in *Kazachstania africana* (Fig 6). Sequence identity between different species is very low–for example, the BLASTP match between the most similar *S*. *cerevisiae* and *Z*. *rouxii* pair has an *E-*value of 0.002 (26% amino acid sequence identity in the aligned region, which is only 123 residues long). To find all the members of the family shown in Fig 6, we used an iterative search procedure in which every member was used individually as a BLASTP query sequence, because many pairs of proteins in the family fail to hit each other (*E* > 10) in BLASTP searches. Characteristically, genes in the family appear as singleton (species-specific or genus-specific) genes in the YGOB database of gene order relationships among Saccharomycetaceae species [61].

Unexpectedly, we found that the members of this gene family tend to be located close to centromeres (Fig 6). In *S*. *cerevisiae*, *YNL018C* and *YNL034W* are located on the left arm of chromosome XIV, at distances of 58 kb and 28 kb from the centromere (*CEN14*). We found a similar pattern of association with centromeres in other Saccharomycetaceae species: 16 of the 18 family members shown in Fig 6 are within 60 kb of a centromere. To assess the statistical significance of this association, we compared the observed median distance of the 8 *Z*. *rouxii* family members to their nearest centromere (18.3 kb) to the distribution of distances seen in 1 million simulations in which sets of eight genes were picked at random from the *Z*. *rouxii* genome. Only 3 of the simulations had a shorter median distance, so we estimate the significance of the observed data to be *P* = 3 x 10^−6^. Similarly, in *K*. *africana* the three family members have a median distance of 21.3 kb from the nearest centromere (empirical *P* = 0.00627 by simulation). Because of this association with centromeric regions, we name the *YNL018C/YNL034W* family the Centroid gene family.

Fig 6 shows only the Centroid family members in species for which complete chromosome sequences are available and centromere locations are known. In addition to these, we also found Centroid family members in the less well characterized genomes of *Candida castellii*, *Nakaseomyces bacillisporus*, *Vanderwaltozyma polyspora*, *Zygosaccharomyces bailii* and *Zygotorulaspora mrakii*. All these species are in the family Saccharomycetaceae. However, there seem to be no Centroid family members in some other Saccharomycetaceae species, such as *Candida glabrata* and *Kluyveromyces lactis*. In total, we identified 28 genes as members of the Centroid family, from species other than *Saccharomyces* (S1 File). Most of these genes are singletons, i.e. they are not at orthologous positions in different genomes. A phylogenetic tree constructed from these sequences is poorly resolved and shows little structure, except that it divides Centroid genes from post-WGD (Whole Genome Duplication) species and non-WGD species into separate clades (S6 Fig). As well as being very divergent in sequence, the Centroid proteins also vary extensively in length (from 212 to 1,291 amino acids), which makes them difficult to align reliably, so we used the motif-finding program MEME [62] to search unaligned Centroid protein sequences for peptide motifs that occur more often than expected by chance. This analysis identified one conserved motif that is present in most members of the family, but at very different locations in different sequences, and two other motifs that are mostly restricted to the non-WGD or post-WGD clades (S7 Fig).

We also searched for homologs of the transmembrane proteins Ynl019c and Ynl033w and found that this protein family is also specific to the family Saccharomycetaceae. In non-*Saccharomyces* species, the *YNL019C/YNL033W* homologs are not found adjacent to Centroid family members, and we did not find any significant association between *YNL019C/YNL033W* homolog locations and centromeres. *YNL019C/YNL033W* homologs are present in some species that do not have Centroid family members, such as *Lachancea kluyveri* (SAKL0B06138g), and conversely they are absent in some Centroid-containing species such as *Nakaseomyces bacillisporus*. Therefore, although the two gene families are both Saccharomycetaceae-specific and sporulation-specific, there is no phylogenetic correlation between the presence of the two families. They are only neighbors in the genome in *Saccharomyces* species.

### Centroid genes are required for sporulation, but their location is unimportant

**Fig 7:**
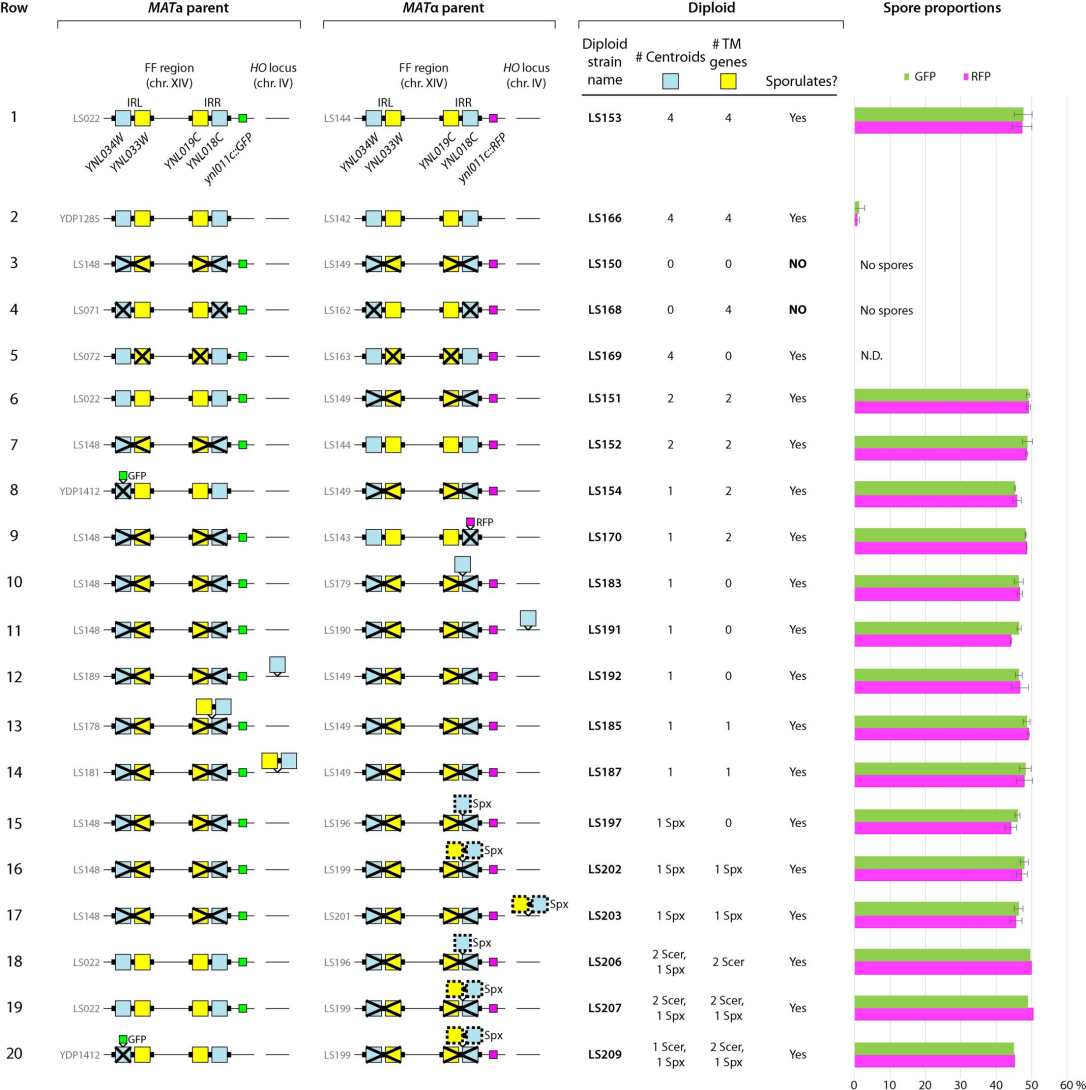
Centroid genes are required for sporulation, but are not meiotic drivers. Each row shows two parents (*MAT***a** and *MAT*α) with genotypes as indicated, that were mated to form a diploid strain that was then tested for sporulation. Blue rectangles represent Centroid genes (*YNL034W*, *YNL018C*), and yellow rectangles represent transmembrane protein genes (*YNL033W*, *YNL019C*). X symbols represent deletions, and gene symbols drawn above the line represent insertions. The “Sporulates?” column indicates ability of the diploid to form tetrad asci on standard sporulation plates (examples are shown in Fig 8), and “NO” indicates that the sporulation rate was <0.2% (no tetrads were seen in >500 cells examined). Bar graphs on the right show the percentages of single spores expressing GFP or RFP, measured by flow cytometry after sporulation of the diploid in liquid media under conditions favoring formation of monad spores (see Materials and Methods). Error bars represent standard deviations of 2–4 replicates where conducted. “Spx” and dashed outlines of genes indicate *S*. *paradoxus* origin. “Scer” indicates *S*. *cerevisiae*, and genes with no label are also from *S*. *cerevisiae*. N.D., not determined.

Since the Centroid (*YNL018C/YNL034W*) and transmembrane protein (*YNL019C/YNL033W*) genes are induced during sporulation, we investigated whether they are essential for this process. We carried out experiments in the *S*. *cerevisiae* Y55 genetic background, which has the INV orientation of the FF region [17,19]. Our strains contain spore-autonomous GFP or RFP fluorescent markers, expressed from the spore-specific *P*_*DIT1*_ promoter, which allow the inheritance of each parental allele to be followed in spores [63,64]. We integrated these markers into the non-essential gene *YNL011C*, which is located between the FF region and the centromere of chromosome XIV, to make parental base strains LS022 (*MAT***a** *ynl011c*::*P*_*DIT1*_*-GFP*) and LS144 (*MAT*α *ynl011c*::*P*_*DIT1*_*-RFP*) (Fig 7, row 1). We then used CRISPR-Cas9 genome editing to make a series of derivatives of these parental strains with deletions of both Centroid genes (blue in Fig 7), deletions of both transmembrane genes (yellow in Fig 7), or deletions of both copies of the whole IR, and crossed them to test the sporulation ability of the resulting diploid. We also made use of strains from Rogers *et al*. [64] in which *YNL034W* or *YNL018C* were disrupted individually by integration of *P*_*DIT1*_*-GFP* or *P*_*DIT1*_*-RFP* (Fig 7, rows 8, 9).

**Fig 8:**
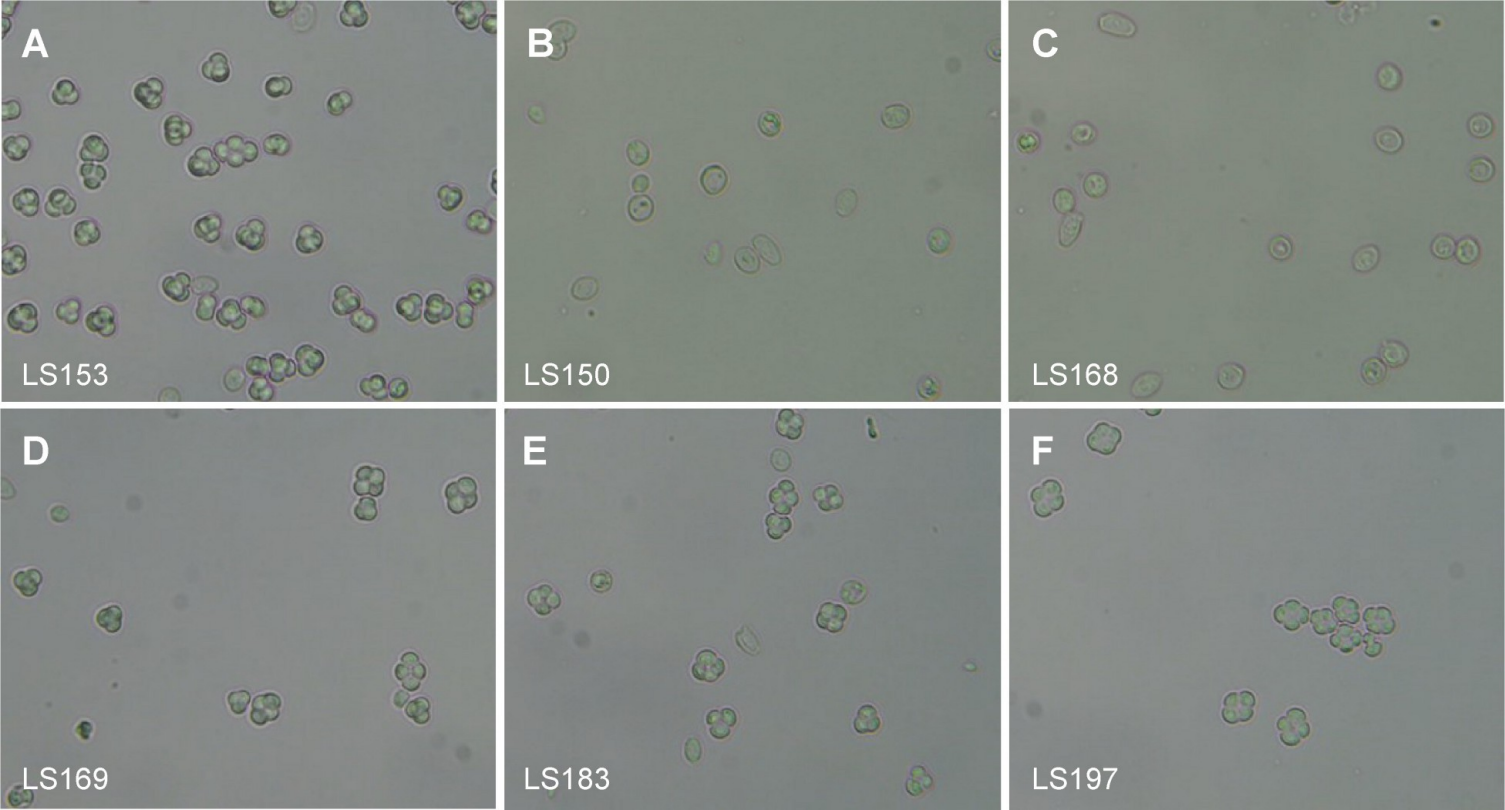
Sporulation phenotypes of *S*. *cerevisiae* diploid strains with and without Centroid genes. (A) Spores are formed in the control strain LS153. (B,C) Strains without the Centroid genes *YNL018C* and *YNL034W* do not form spores. No tetrads were seen in >500 cells examined from each of LS150 and LS168. (D) Spores are formed in strain LS169 lacking the transmembrane protein genes *YNL019C* and *YNL033W*. (E,F) Spores are formed in strains in which both IRs were deleted and a Centroid gene (*YNL018C*) was reintroduced from either *S*. *cerevisiae* (LS183) or *S*. *paradoxus* (LS197).

We found that diploids with no Centroid genes were completely unable to sporulate (Fig 7, rows 3, 4 and Fig 8A–8C), whereas all diploids containing at least one Centroid gene sporulated normally. A single Centroid gene at either the *YNL018C* locus or the *YNL034W* locus of one parent is sufficient for sporulation (Fig 7, rows 8–9). The transmembrane protein genes are not required for sporulation (Fig 7, row 5 and Fig 8D). We also reintroduced genes into parental strains lacking both copies of the whole IR. Reintroducing a single *S*. *cerevisiae* Centroid gene either close to the former IR region on chromosome XIV, or at the *HO* locus on chromosome IV, restores sporulation (Fig 7, rows 10–12 and Fig 8E). In addition, introducing a heterologous Centroid gene from *S*. *paradoxus* with its native promoter, or the *S*. *paradoxus* Centroid-transmembrane gene pair (*YNL018C-YNL019C*), enables strains lacking *S*. *cerevisiae* Centroid genes to sporulate (Fig 7, rows 15–17 and Fig 8F).

These experiments show that Centroid genes play an essential role in sporulation. Even a single hemizygous Centroid gene located anywhere in a diploid genome is sufficient for sporulation. It does not need to be duplicated, and it does not need to be located near the FF region on chromosome XIV.

### No evidence for meiotic drive by Centroid genes

We hypothesized that the Centroid gene family might be undergoing meiotic drive, because its members are consistently located close to centromeres and are evolving rapidly, and (in *Saccharomyces* species) they are associated with an inversion polymorphism. Some of these features are seen in meiotic drive systems in other organisms [65,66]. For example, the poison and antidote genes of the *Neurospora intermedia Sk-2* spore killer system are rapidly evolving, centromere-linked, and located in a region of the genome in which recombination is suppressed by inversions [67].

To test the hypothesis that Centroid genes are meiotic drivers, we quantified the inheritance of the two parental haplotypes of the FF region in crosses between *S*. *cerevisiae* strains with different Centroid genotypes. Diploids were sporulated in liquid media in conditions favoring the formation of monad asci [68], and the numbers of single spores inheriting the *P*_*DIT1*_*-GFP* or *P*_*DIT1*_*-RFP* fluorescent markers were counted by flow cytometry (Fig 7 bar charts, and S8 Fig). We scored approximately 50,000 spores per cross. In a control cross in which both parents are wildtype at the Centroid loci, we observed approximately equal numbers of spores expressing GFP and RFP, as expected under Mendelian inheritance (Fig 7, row 1). However, we also observed equal numbers of GFP and RFP spores in every cross in which Centroid genes were disrupted, including crosses in which the copy of chromosome XIV from one parent contained no Centroid genes and the other copy contained either two Centroid genes (Fig 7, rows 6–7) or one Centroid gene (rows 8–10, 13). We also found equal numbers of GFP and RFP spores in crosses in which one parent carried a Centroid gene from *S*. *paradoxus* and the other parent had either no Centroid gene (rows 15–16) or *S*. *cerevisiae* Centroid gene(s) (rows 18–20). Therefore, these experiments fail to support our hypothesis and we conclude that Centroid genes are not meiotic drivers in *S*. *cerevisiae*.

## Discussion

It is surprising that the REF/INV inversion polymorphism in *S*. *cerevisiae* remained undetected during decades of gene mapping and analysis of the meiotic recombination landscape in this model organism. Even though the IRs were discovered in 1997 and the first INV strain was reported in 2007 [14,15], the inversion has continued to be overlooked in the genome assemblies of many INV strains because the IRs are too large and too similar to be resolved separately by standard short-read next-generation sequencing methods, whereas they are resolved by long-read methods. The inversion polymorphism creates a non-recombining block of 24 kb near *CEN14* that segregates as a single unit in meiosis, but only in crosses between strains with opposite orientations. The fact that no quantitative trait loci have been mapped to this block in the BY x RM cross and other crosses of REF x INV genetic backgrounds suggests that the two orientations of the FF region do not affect phenotypes in any significant way [38–41]. There are no origins of replication inside the FF region, so its orientation is not expected to affect the overall replication profile of chromosome XIV. However, the orientation does affect the position of genes inside the FF region relative to the replication origins located beyond the IRs on each side (*ARS1423* and *ARS1424*; Fig 1), which may affect the timing of replication of genes such as the histone gene pair *HHT2-HHF2*.

An identical arrangement of two 4.2-kb IRs exists in all eight species of the genus *Saccharomyces*, with essentially the same boundaries in every species. We found phylogenetic evidence that the IRs are being homogenized within species and, to a lesser extent, within strains (Fig 5). We suggest that the molecular mechanism of homogenization of the IRs is gene conversion during non-allelic homologous recombination (NAHR) between IRL and IRR [11]. Gene conversion during NAHR will tend to make IRL and IRR identical in sequence, because it uses one of them as a template to repair a double-strand break in the other [69]. About 4% of gene conversion events are resolved as crossovers [69], and if a crossover occurs during NAHR between sequences that are in opposite orientations, the region between them will become inverted [11,70]. We propose that continual gene conversion between the IRs has had the effect of continually inverting the FF region within each *Saccharomyces* species. This mechanism is the same as one proposed by Cáceres *et al*. [11] for the maintenance of a recurrent inversion, flanked by homogenized IRs, on the X chromosome of many mammalian species.

Homogenization of the IRs has the consequence that (for example) *S*. *cerevisiae YNL018C* is more similar to its paralog *S*. *cerevisiae YNL034W* than to its ortholog *S*. *paradoxus YNL018C* –they have 97% and 72% amino acid sequence identity respectively. Similar homogenization occurs in some other *Saccharomyces* gene families, such as histone genes and ribosomal protein genes [71], and it is generally thought that these genes are duplicated because high expression of their protein products is needed, and that they are homogenized because sequence variation of their protein products is deleterious [72]. The genes located in the IR are highly transcribed during sporulation, so their protein products may be abundant. However, sequence variation in Centroid genes seems to be tolerated, because diploids carrying both *S*. *cerevisiae* and *S*. *paradoxus* Centroid genes sporulate normally (Fig 7, rows 18–20).

*YNL018C* and *YNL034W* are the founding members of a diverse gene family, Centroid, which is present only in Saccharomycetaceae species. The family has the unusual property that most of its members are located close to centromeres. Only two Centroid genes (*ZYRO0A10868g* on *Z*. *rouxii* chromosome 1, and *TBLA0I01200* on *T*. *blattae* chromosome 9) are more than 60 kb from a centromere (Fig 6), and for both of them we can infer by comparing gene order relationships [61] that they were originally within this range but have been moved away from the centromere by genomic rearrangements. *ZYRO0A10868g* maps to a position near an ancestral centromere site (*Anc_CEN5*, corresponding to *Z*. *rouxii CEN2*; [73]), and we can infer that it was originally located about 57 kb from this centromere until an interchromosomal rearrangement in *Z*. *rouxii* moved it to its current location (breakpoint between genes *SAG1/ZYRO0A11462g/Anc_5*.*225* and *YJR003C/ZYRO0B09944g/Anc_5*.*224*). Similarly, *TBLA0I01200* also maps to a position near an ancestral centromere site (*Anc_CEN2*, corresponding to *T*. *blattae CEN9*), and we can infer that it was located about 56 kb from this centromere until a rearrangement within *T*. *blattae* chromosome 9 moved it away (breakpoint between *VPS20/TBLA0I01100/Anc_2*.*492a* and *YAP3/TBLA0I00300/Anc_2*.*493*). Thus most of the Centroid genes are located within, or just outside, the 30–50 kb pericentric region around each centromere that is enriched in cohesin and condensin and forms a bottle-brush structure [74,75].

As in other eukaryotes, the chromosomal location of most genes in budding yeasts seems to be arbitrary and unrelated to their function or other properties [76]. Only a few examples of non-random gene locations are known, such as the metabolic clusters of *GAL* and *DAL* genes [77,78]. A few gene families such as the *MAL* and *COS* genes are specific to subtelomeric regions [79,80]. However, as far as we are aware, the only genes that have a preference for pericentromeric locations, in any eukaryote, are mobile genetic elements. In several yeast species, retrotransposons of the Ty5 superfamily are found only near the centromere of each chromosome and occur in clusters–for example in *Scheffersomyces stipitis* [81,82] and *Ogataea polymorpha* [83]. This location bias probably results from an interaction between the retrotransposon’s integrase and a centromere-binding protein [84]. Retrotransposons code for well-conserved protein domains and are readily identifiable across all eukaryotes. In contrast, Centroid genes are highly divergent and lack homologs outside the Saccharomycetaceae, and there is no indication (such as evidence of recent transposition) that they are mobile elements.

The FF region and its neighboring IRs are a very unusual region of the *S*. *cerevisiae* genome. We have found that the inversion polymorphism is ancient and recurrent, and that the FF region inverts because the IRs are continually being homogenized. There appears to be evolutionary pressure to homogenize the IRs, because they have been maintained as homogeneous for millions of years, but we have not identified the source of this pressure. We found that one of the genes in the IR, Centroid (*YNL018C/YNL034W*), is essential for sporulation but we do not know why the Centroid gene family is non-randomly located near centromeres in Saccharomycetaceae species. We ruled out meiotic drive as a possible explanation for the Centroid family’s unusual properties. Our work has provided more understanding of some aspects of the FF region and Centroid genes but leaves many questions unanswered.

## Materials and methods

### Bioinformatics

References and NCBI accession numbers for the long-read genome assemblies of *Saccharomyces* species analyzed in this study are given in S1 Table.

For the analysis in Fig 3, genotype data for the segregants in each cross was downloaded from the supplementary information of the original publications [29–35] and crossover sites were mapped by reference to the parental genotypes at each SNP site.

For the analysis in Fig 4, SGRP genome sequence assemblies of chromosome XIV from Liti *et al*. [22] were aligned individually to a reference sequence (S288C for *S*. *cerevisiae*, CBS432 for *S*. *paradoxus*) of chromosome XIV using MUMmer [85]. Nucleotide substitutions and deletions (but not insertions) relative to the reference identified in each alignment were then written onto the reference sequence to generate a pseudochromosome sequence for each strain, identical in length to the reference for that species, which were then collated to make multiple sequence alignments (MSAs). Four MSAs (*i*.*e*., the FF region of chromosome XIV, one for each species, and the remaining sites on chromosome XIV, one for each species) were then used for phylogenetic analysis. For each MSA, the optimal model of sequence evolution (SE) was identified using ModelFinder [86], with the –mtree option invoked, and the optimal maximum-likelihood (ML) tree was identified using IQ-TREE 2 [87], given the optimal model of SE.

For the analysis in Fig 5, long-read sequence assemblies were taken from the sources listed in S1 Table. DNA sequences of the region between the 3’ end of tRNA-Ile and the stop codon of *YNL019W/YNL033C* were aligned using MUSCLE [88] as implemented in Seaview 5.0.4 [89]. The optimal model of SE was identified using ModelFinder [86], with the –mtree option invoked, and the optimal ML tree was identified using IQ-TREE 2 [87], given the optimal model of SE. We excluded sequences of Chinese strains from Bendixsen *et al*. [17] because their IR regions contain deletions that suggest they may be misassembled.

For the phylogeny in S6 Fig, amino acid sequences were aligned using MAFFT [90] with the einsi option invoked. Using AliStat [91], the MSA was found to be 26.1% complete (*i*.*e*., it has an abundance of missing data). Accordingly, we masked the MSA so that sites with over 50% missing data were removed. This yielded a shorter MSA of 389 sites, with a completeness of 77.2%. We then surveyed the MSA to measure the strength of historical signal. Using SatuRation (https://github.com/lsjermiin/SatuRation.v1.0), we found it to be between 0.000 and 0.977, implying a strong signal (*i*.*e*., 0.0) for some sequences and an extremely eroded signal for others (on a scale of 0 to 1). Therefore, we used maximum likelihood to identify the optimal model of sequence evolution and, using this model, to identify the optimal phylogeny. Using ModelFinder [86], with the –mtree option invoked, the VT+F0+R3 model was found to yield the best fit between tree, model and data (using BIC). Using IQ-TREE 2 [87], the optimal tree was identified, and using the UFBoot2 method [92] bootstrap support scores were computed for internal edges in the tree.

The analysis in S7 Fig was done using the MEME webserver (https://meme-suite.org/meme) with these options: meme sequences.fa -protein -oc .-nostatus -time 14400 -mod anr -nmotifs 3 -minw 6 -maxw 50 -objfun classic -markov_order 0

### PCR assays of FF region orientation

Sources of strains used in PCR assays are given in S1 Table. After cultivation, cells were pelleted and genomic DNA extracted using phenol/chloroform/isoamyl alcohol (25:24:1). Genomic DNAs were then diluted to 100 ng/μL in water, and 1 μL was used per PCR reaction. PCR screening of the FF region orientation was done using the primers listed in S3 Table. The PCR program consisted of: 95°C 1 min; 25 x (95°C 30 s; 65°C 30 s; 72°C 5 min); 72°C 10 min. For *S*. *cerevisiae*, PCR amplification was done using Phusion Taq polymerase (Thermo Fisher) and the primer pairs ScUf2/ScUr2 (REF), ScDf2/ScDr2 (REF), ScUf2/ScDf2 (INV), and ScDr2/ScUr2 (INV). For *S*. *paradoxus*, PCR amplification was done using Q5 Taq polymerase (New England Biolabs) and the primer pairs ScUf/ScUr2 (REF), ScDf2/ScDr (REF), ScUf/ScDf2 (INV), and ScDr/ScUr2 (INV). For *S*. *uvarum*, PCR amplification was done using Q5 Taq polymerase (New England Biolabs) and the primer pairs SuUf/SuUr (REF), SuDf/SuDr (REF), SuUf/SuDf (INV), and SuDr/ScUr (INV). 1 μL of the PCR reactions were then analyzed by 1% agarose gel electrophoresis (+ GelRed 0.5 X), at 100 V for 30 to 60 min. Genotypes were scored as REF or INV when at least one of the specific REF or INV primer pairs allowed the amplification of a product of the expected size, with no unspecific amplifications of other sizes.

### Strain construction

All strains were constructed in *S*. *cerevisiae* Y55 haploid prototrophs, which were then mated to create diploid strains. Strains are listed in S4 Table, and oligonucleotides used during strain construction are listed in S5 Table.

All strains were transformed using a standard lithium acetate protocol [93]. The presence of both *MAT*a/*MAT*α in diploid strains was verified by colony PCR [94]. Strains LS022/LS023 were generated by transforming strains YDP1285/YDP1307 with a linear DNA fragment amplified using primers #51/52 and genomic DNA from YDP1351/YDP1343 (*P*_*DIT1*_*-GFP_URA3/ P*_*DIT1*_*-RFP_LEU2*). Strains LS142, LS143, and LS144 were created by transforming strains YDP1307, YDP1399, and LS023, respectively, with a linear DNA fragment amplified using primers #113/114 and plasmid pIL75 [95]. Centroid/TM deletion and complementation strains were constructed using a two-plasmid CRISPR-Cas9 system plus linear repair templates. Parental strains were first transformed with the Cas9-NAT plasmid, which has Cas9 under a constitutive *TEF1* promoter, with a NatMX selectable marker (https://www.addgene.org/64329/). Transformants were selected on YPD plates containing 100 μg/mL nourseothricin (NTC; HKI Jena). Cells containing the Cas9-NAT plasmid were then transformed with a guide plasmid plus linear repair template(s), and transformants were selected on YPD plates containing 100 μg/mL NTC plus 300 μg/mL hygromycin (Hyg; Invitrogen). Transformants containing the correct mutation(s) (determined by colony PCR), were cured of both plasmids by serial passages to YPD plates.

The inserts for CRISPR guide RNA plasmids pJBo9-18C/34W, pJBo9-19C/33W, pJBo9-18C_comp, and pJBo9-HO_comp were amplified using primers #55/5, #60/5, #99/5, and #172/5, respectively, and pJBo9 ([96]; gift from Prof. Meru Sadhu) as the template. The resulting fragments were cloned into *Kpn*I/*BstE*II digested, SAP treated pJBo9 by Gibson assembly (NEB). Deletion repair templates were generated by primer extension, and complementation repair templates were amplified using genomic DNA from strain YDP1351 (*S*. *cerevisiae*) or CBS432 (*S*. *paradoxus*).

Strains LS071/LS162 (*Δynl018c/Δynl034w*) were created by transforming strains LS022/LS144, respectively, with pJBo9-18C/34W plus a repair template amplified using primers #94/95.

Strains LS072/LS163 (*Δynl019c/Δynl033w*) were created by transforming strains LS022/LS144, respectively, with pJBo9-19C/33W plus a repair template amplified using primers #96/97.

Strains LS148/LS149 (ΔIRs) were created by transforming strains LS022/LS144, respectively, with pJBo9-18C/34W plus a mix of repair templates amplified using primers #120/121 and #128/129.

Strain LS178 (*S*. *cerevisiae YNL018C/19C* complemented at original locus) was created by transforming strain LS148 with pJBo9-18C_comp plus a repair template amplified using primers #167/171.

Strain LS179 (*S*. *cerevisiae YNL018C* complemented at original locus) was created by transforming strain LS149 with pJBo9-18C_comp plus a repair template amplified using primers #167/169.

Strain LS181 (*S*. *cerevisiae YNL018C/19C* complemented at *HO* locus) was created by transforming strain LS148 with pJBo9-HO_comp plus a repair template amplified using primers #174/176.

Strains LS189/190 (*S*. *cerevisiae YNL018C* complemented at *HO* locus) were created by transforming strains LS148/LS149, respectively, with pJBo9-HO_comp plus a repair template amplified using primers #174/175.

Strain LS196 (*S*. *paradoxus YNL018C* complemented at original locus) was created by transforming strain LS149 with pJBo9-18C_comp plus a repair template amplified using primers #181/182.

Strain LS199 (*S*. *paradoxus YNL018C/19C* complemented at original locus) was created by transforming strain LS149 with pJBo9-18C_comp plus a repair template amplified using primers #181/183.

Strain LS201 (*S*. *paradoxus YNL018C/19C* complemented at *HO* locus) was created by transforming strain LS149 with pJBo9-HO_comp plus a repair template amplified using primers #184/186.

### Sporulation

Diploid strains were inoculated into 5 mL of YP plus 1% potassium acetate (Sigma-Aldrich) and grown overnight at 30°C with shaking. For tetrad enrichment, 1 mL of the overnight culture was harvested by centrifugation (0.4 *g* × 3 min at room temperature), washed twice with 1 mL dH_2_O (0.4 *g* × 3 min at room temperature), and finally resuspended in 2 mL 2% potassium acetate in a 50 mL Falcon tube. Monad enrichment was performed similarly but with the following changes: 2 mL of overnight culture were harvested, and cells were finally resuspended in 10 mL of dH_2_O in a 250 mL Erlenmeyer flask. The cells were then incubated for 48 h at 30°C with shaking. Harvested spores were stored at 4°C.

### Flow cytometry sample preparation and analysis

Monad spore samples (200 μL) were centrifuged at 21,000 *g* × 1 min, and the pellets were resuspended in 50 μL of 0.5 mg/mL Zymolyase 100T (USBiological) in 0.1 M sorbitol (Sigma-Aldrich), and incubated at 30°C overnight (statically). Next, 450 μL 1.5% IGEPAL (Sigma-Aldrich) were added to the samples and then vortexed for 2 min. The spore samples were then sonicated at maximum power for 20 min in a sonicating water bath (Langford Ultrasonic Bath, Model 500) with ice. The samples were then spun down at 21,000 *g* × 1 min, washed twice with 200 μL dH_2_O, resuspended in 200 μL dH_2_O, and vortexed for 2 min. The samples were then spun down at 21,000 *g* × 1 min, resuspended in 200 μL 1.5% IGEPAL, and sonicated at maximum power for 20 min in sonicating water bath with ice. Processed samples were stored at 4°C prior to FC analysis.

Samples were analyzed using a Beckman Coulter CytoFLEX LX flow cytometer and CytExpert software. An initial scatter gate (FSC-H vs SSC-H) was applied to remove debris and noise. Next, a combination of gates was applied to select for single events (singlets; FSC-H vs FSC-width, FSC-A vs FSC-H, and FSC-H vs SSC-H [for separation of monad and dyad spores]). A further gate was applied to remove spore autofluorescence “noise” (NUV450-A vs V525-KrO-A). GFP/RFP expression in the spores was detected using GFP B525-FITC-H/Tomato Y585-PE-H. The position of the gate was determined based on a wild type control that does not contain GFP or RFP (strain LS166). A minimum of 50,000 events were analyzed per sample, and no compensation was applied (S8 Fig).

## Supporting information

S1 Fig PCR assays of FF region orientation in *Saccharomyces* species. (A) *S*. *cerevisiae*. (*i*). *S*. *cerevisiae* SGRP strains. These strains are diploid; strains with “/A” after the OS (“original strain”) name are monosporic isolates, whereas the others were not sporulated [22]. (*ii*) and (*iii*). Diploid natural isolates of *S*. *cerevisiae*. These isolates were used in previous studies that measured their heterozygosity at SNP sites, either by whole-genome sequencing (Magwene *et al*. [25]) or by sequencing about 1% of the genome by RAD-seq (Cromie *et al*. [26]). The strains are listed in decreasing order of the numbers of heterozygous sites found in those studies. (*iv*). Schematic showing the locations of the PCR primer pairs used in FF region orientation assays. Amplification of PCR products 1 and 2 indicates REF orientation, and amplification of PCR products 3 and 4 indicates INV orientation. (B) *S*. *paradoxus*, monosporic isolates from SGRP [22]. (C) *S*. *uvarum*, natural isolates, presumed to be diploid [24].
(PDF)

S2 Fig Synteny relationships around the FF region, based on information in the Yeast Gene Order Browser [61]. Genes in the FF region on *S*. *cerevisiae* chromosome XIV are named in red. Dots represent protein-coding genes, and triangles represent tRNA genes with orientations as shown. Homologous genes (orthologs, or pairs of paralogs in post-WGD species) are drawn in the same row. The three “Non-WGD species” columns in the center show the order of genes in two species that did not undergo WGD (*Lachancea kluyveri* and *Zygotorulaspora mrakii*), and in the inferred last common ancestor of the WGD clade [97]. This gene order is compared to two post-WGD species: *S*. *cerevisiae* and *Candida castellii* [98]. Each post-WGD species has two paralogous chromosomal regions (Track A and Track B), corresponding to parts of chromosomes XIV and IX/IV in *S*. *cerevisiae*, and parts of scaffolds s05 and s21/s27 in *C*. *castellii*. The FF region of *S*. *cerevisiae* is drawn in INV orientation, and its gene order shows similarities with the non-WGD species, with *C*. *castellii* Track A, and with Track B from both of the post-WGD species. *YNL018C/YNL034W* and *YNL019C/YNL033W* do not have orthologs in any of the other species shown, but a pair of tRNA-Ile genes in opposite orientations forms a small IR in the non-WGD species and in *S*. *cerevisiae* Track B (chromosome IX), as shown by the gray shading. The species shown were chosen because they have relatively little rearrangement in this area of the genome. One inversion shared by *S*. *cerevisiae* and *C*. *castellii*, and one inversion unique to *S*. *cerevisiae*, are visible. A few species-specific genes have been omitted for clarity, and the noncoding RNA genes *NME1* and *SNR66* are not shown because they are not annotated in all genomes.
(PDF)

S3 Fig A single crossover inside the FF region in a REF x INV cross would result in gross chromosomal defects. Progeny chromosomes are hairpins (isochromosomes) with either no centromere or two centromeres.
(PDF)

S4 Fig PCR assays of FF region orientation in *S*. *cerevisiae* strains from Nishant *et al*. [48]. The parental strain EAY2531 is a diploid SK1 derivative (INV/INV), and all other strains are derived from EAY2531. The 20 strains EAY2823 to EAY2842 are independent lines that went through approximately 1,740 vegetative generations each. The 19 strains EAY2804 to EAY2822 are independent lines that went through approximately 1,000 vegetative generations and 50 meiotic generations each. Despite the high number of generations in both cases, no inversion of the FF region was detected.
(PDF)

S5 Fig Transcription of genes located in the *S*. *cerevisiae* IRs, in public RNAseq datasets. Green tracks indicate rightward transcription, and red tracks indicate leftward transcription. (A) Meiosis and sporulation time course from Brar *et al*. [55]. Their study combined two time courses (a “traditional meiosis time course” with points A-V, and a time course after artificial induction of the late meiosis regulator *NDT80*, with points 1–18), into a single master time course with points in the order shown. To save space, data from every second time point is shown. Data from NCBI SRA accession numbers SRR387838 to SRR387870. (B) Meiosis time course data from Gould *et al*. [56] after induction of *NDT80*. In strain SK1, IRL is interrupted by a Ty3 element [51]. Data from NCBI SRA accession numbers SRR2831307 to SRR2831321. (C) Transcription during vegetative exponential growth of S288C wildtype haploid cells. Data from Wery *et al*. [57], NCBI SRA accession number SRR2045245.
(PDF)

S6 Fig Phylogenetic tree of all Centroid proteins. The tree was inferred as described in Materials and Methods. Circled numbers indicate pairs or trios of genes that have orthologous (syntenic) genomic locations [61].
(PDF)

S7 Fig Conserved amino acid motifs in Centroid proteins. (A) Locations of the motifs within Centroid proteins. (B) Sequence logos of the identified motifs. Unaligned sequences of 29 Centroid sequences from Saccharomycetaceae species were analyzed using MEME [62] to identify amino acid sequence motifs that occur more often than expected by chance. The top 3 most statistically significant motifs are shown. Motif 1 is present in almost every sequence, whereas Motifs 2 and 3 are largely restricted to non-WGD and post-WGD species, respectively. To avoid an over-representation of *Saccharomyces* sequences, only *S*. *cerevisiae* Ynl018c was included from this genus.
(PDF)

S8 Fig Gating strategy for flow-cytometry analysis. An initial scatter gate was used to remove debris and noise (FSC-H vs SSC-H). Single events (vs. aggregates) were then selected using a combination of gates (FSC-H vs FSC-Width and FSC-A vs FSC-H). The singlets were then separated into monads and dyads/tetrads (FSC-H vs SSC-H). The monad population was then gated for auto-fluorescence “noise” (NUV450-A vs V525-KrO-A). The gates for GFP (B525-FITC-H) and RFP (Y585-PE-H) expression were then set based on the expression profile of the negative control population (strain LS166). Percent expression in each quadrant was then scored. This example shows strain LS153, with 49.34% of monads scored as expressing only *P*_*DIT1*_*-GFP* (GFP+ RFP-) and 48.75% scored as expressing only *P*_*DIT1*_*-RFP* (GFP- RFP+).
(PDF)

S1 Table Orientation of the FF region in strains of *Saccharomyces* species, determined by PCR assays and/or long-read sequencing. PCR assays were conducted for this study. Gel images are shown in S1 Fig.
(XLSX)

S2 Table FF region orientations in 24 natural isolates of *S*. *cerevisiae* from Taiwan. Sequence data are from Lee *et al*. [23].
(PDF)

S3 Table PCR primer sequences for FF region amplification. (PDF)

S4 Table Genetically modified *S*. *cerevisiae* strains used in this study. (PDF)

S5 Table Oligonucleotides used for strain construction. (PDF)

S1 Data Flow cytometry numerical data for Fig 7. (XLSX)

S1 File Amino acid sequences of Centroid family members (Text file with amino acid sequnces in FASTA format). (TXT)
