# Supplementary material for: A widespread inversion polymorphism conserved among Saccharomyces species is caused by recurrent homogenization of a sporulation gene family
Source: PLoS Genet. 2022 Nov 28;18(11):e1010525. doi: 10.1371/journal.pgen.1010525 (PMC9731477; doi:10.1371/journal.pgen.1010525)
Supplement: S1 Fig — (A) S. cerevisiae. (i). S. cerevisiae SGRP strains. These strains are diploid; strains with “/A” after the OS (“original strain”) name are monosporic isolates, whereas the others were not sporulated [22]. (ii) and (iii). Diploid natural isolates of S. cerevisiae. These isolates were used in previous studies that measured their heterozygosity at SNP sites, either by whole-genome sequencing (Magwene et al. [25]) or by sequencing about 1% of the genome by RAD-seq (Cromie et al. [26]). The strains are listed in decreasing order of the numbers of heterozygous sites found in those studies. (iv). Schematic showing the locations of the PCR primer pairs used in FF region orientation assays. Amplification of PCR products 1 and 2 indicates REF orientation, and amplification of PCR products 3 and 4 indicates INV orientation. (B) S. paradoxus, monosporic isolates from SGRP [22]. (C) S. uvarum, natural isolates, presumed to be diploid [24]. (PDF) [file pgen.1010525.s001.pdf]

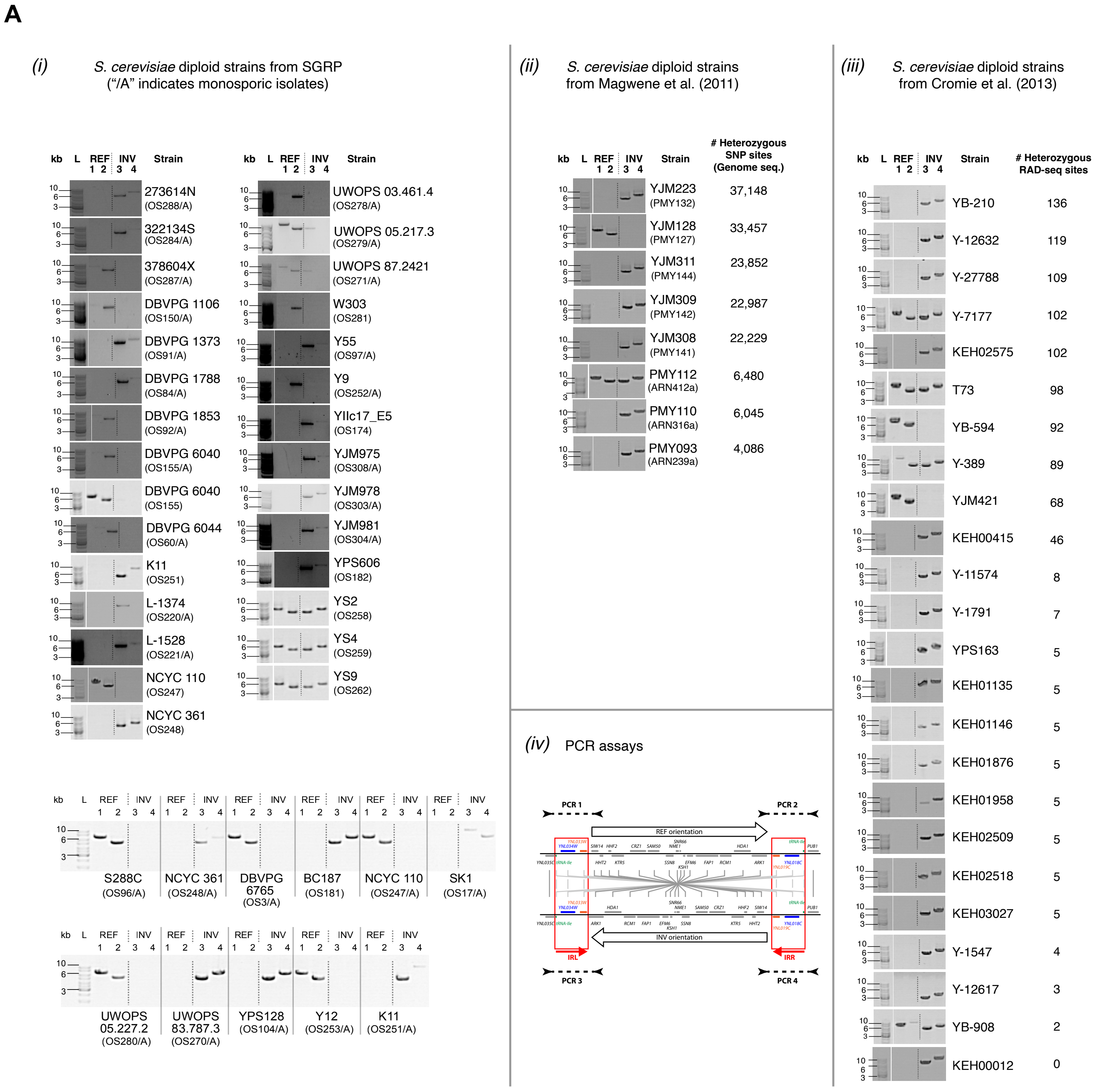
A
(i) S. cerevisiae diploid strains from SGRP
("/A" indicates monosporic isolates)
kb L REF 1 2 INV 3 4 Strain
273614N (OS288/A)
322134S (OS284/A)
378604X (OS287/A)
DBVPG 1106 (OS150/A)
DBVPG 1373 (OS91/A)
DBVPG 1788 (OS84/A)
DBVPG 1853 (OS92/A)
DBVPG 6040 (OS155/A)
DBVPG 6040 (OS155)
DBVPG 6044 (OS60/A)
K11 (OS251)
L-1374 (OS220/A)
L-1528 (OS221/A)
NCYC 110 (OS247)
NCYC 361 (OS248)
UWOPS 03.461.4 (OS278/A)
UWOPS 05.217.3 (OS279/A)
UWOPS 87.2421 (OS271/A)
W303 (OS281)
Y55 (OS97/A)
Y9 (OS252/A)
YIIc17_E5 (OS174)
YJM975 (OS308/A)
YJM978 (OS303/A)
YJM981 (OS304/A)
YPS606 (OS182)
YS2 (OS258)
YS4 (OS259)
YS9 (OS262)
S288C (OS96/A)
NCYC 361 (OS248/A)
DBVPG 6765 (OS3/A)
BC187 (OS181)
NCYC 110 (OS247/A)
SK1 (OS17/A)
UWOPS 05.227.2 (OS280/A)
UWOPS 83.787.3 (OS270/A)
YPS128 (OS104/A)
Y12 (OS253/A)
K11 (OS251/A)
(ii) S. cerevisiae diploid strains from Magwene et al. (2011)
kb L REF 1 2 INV 3 4 Strain # Heterozygous SNP sites (Genome seq.)
YJM223 (PMY132) 37,148
YJM128 (PMY127) 33,457
YJM311 (PMY144) 23,852
YJM309 (PMY142) 22,987
YJM308 (PMY141) 22,229
PMY112 (ARN412a) 6,480
PMY110 (ARN316a) 6,045
PMY093 (ARN239a) 4,086
(iii) S. cerevisiae diploid strains from Cromie et al. (2013)
kb L REF 1 2 INV 3 4 Strain # Heterozygous RAD-seq sites
YB-210 136
Y-12632 119
Y-27788 109
Y-7177 102
KEH02575 102
T73 98
YB-594 92
Y-389 89
YJM421 68
KEH00415 46
Y-11574 8
Y-1791 7
YPS163 5
KEH01135 5
KEH01146 5
KEH01876 5
KEH01958 5
KEH02509 5
KEH02518 5
KEH03027 5
Y-1547 4
Y-12617 3
YB-908 2
KEH00012 0
(iv) PCR assays
PCR 1
PCR 2
REF orientation
INV orientation
IRL
IRR
PCR 3
PCR 4

S1A Figure

B

S. paradoxus strains from SGRP (monosporic isolates)

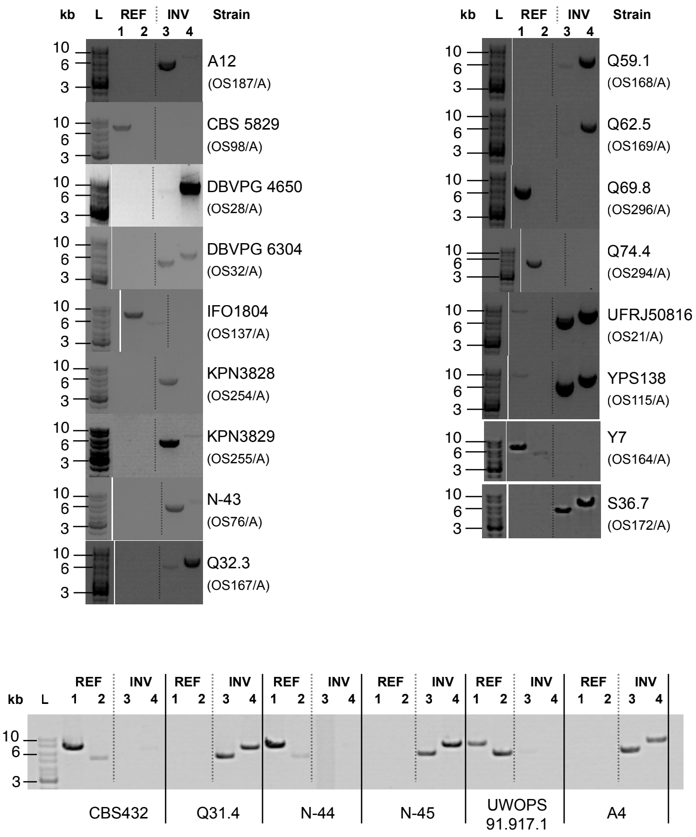

C

S. uvarum natural isolates

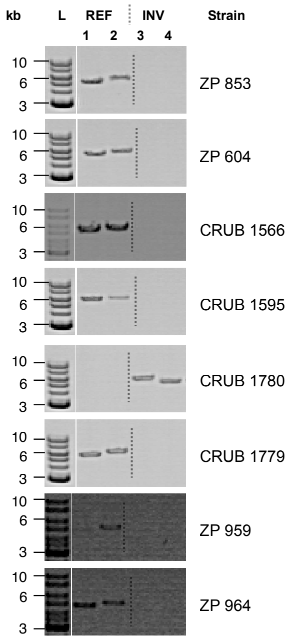

S1B,C Figure
