# Supplementary figures and images for: A widespread inversion polymorphism conserved among Saccharomyces species is caused by recurrent homogenization of a sporulation gene family
Source: PLoS Genet. 2022 Nov 28;18(11):e1010525. doi: 10.1371/journal.pgen.1010525 (PMC9731477; doi:10.1371/journal.pgen.1010525)

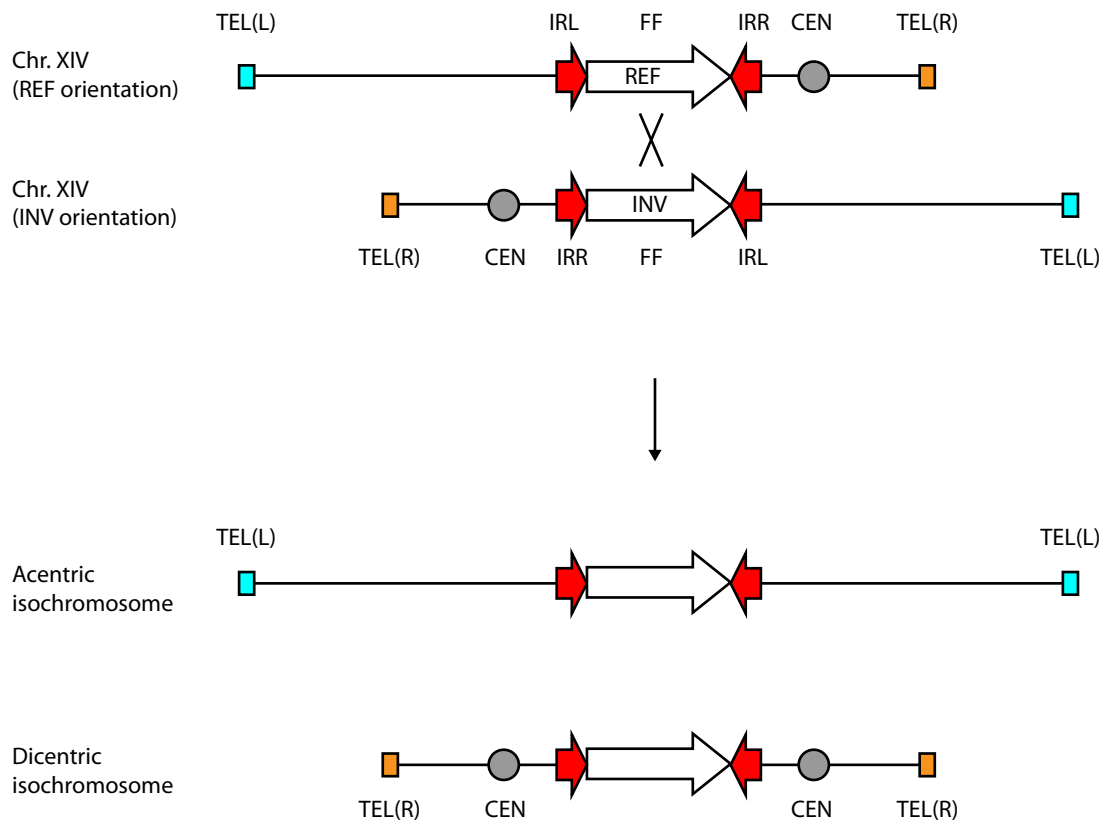

S3 Figure

Supplement: S3 Fig — Progeny chromosomes are hairpins (isochromosomes) with either no centromere or two centromeres. (PDF) [file pgen.1010525.s003.pdf]

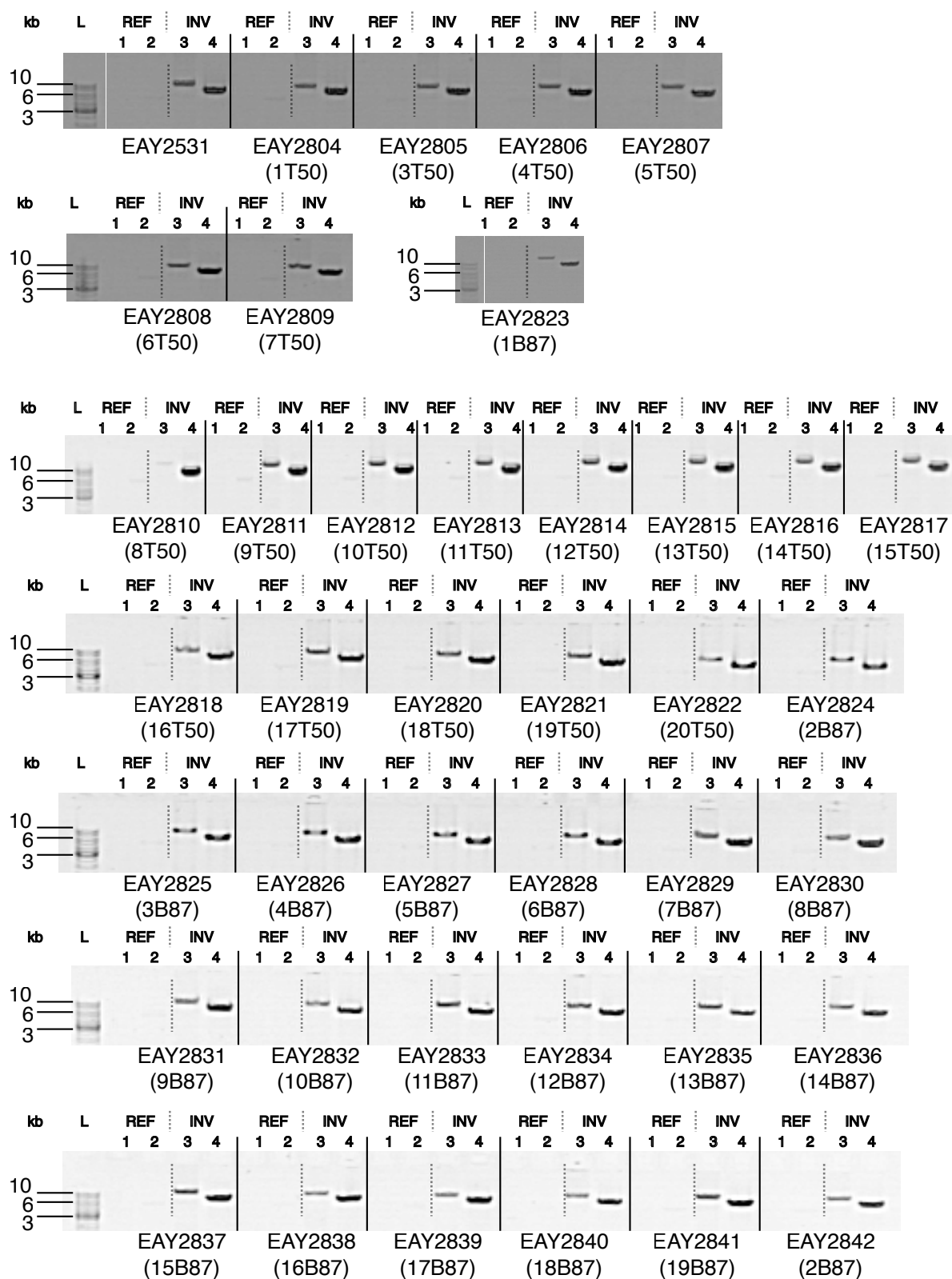

S4 Figure

Supplement: S4 Fig — The parental strain EAY2531 is a diploid SK1 derivative (INV/INV), and all other strains are derived from EAY2531. The 20 strains EAY2823 to EAY2842 are independent lines that went through approximately 1,740 vegetative generations each. The 19 strains EAY2804 to EAY2822 are independent lines that went through approximately 1,000 vegetative generations and 50 meiotic generations each. Despite the high number of generations in both cases, no inversion of the FF region was detected. (PDF) [file pgen.1010525.s004.pdf]

A

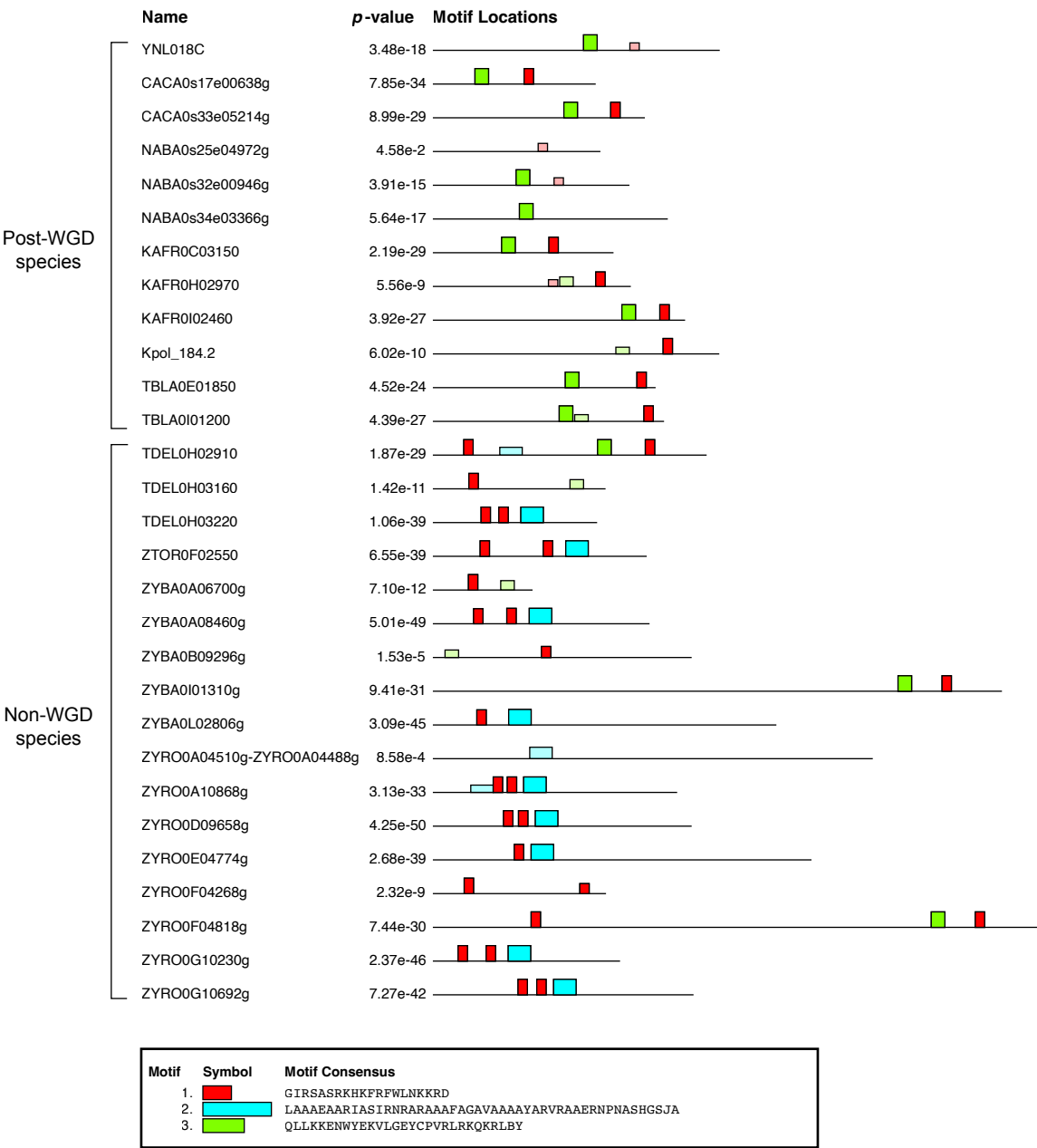

B

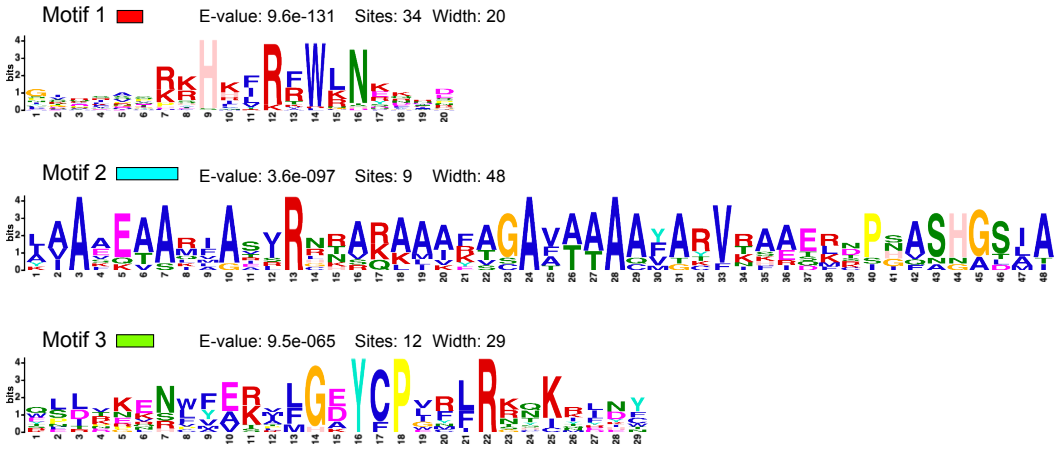

Supplement: S7 Fig — (A) Locations of the motifs within Centroid proteins. (B) Sequence logos of the identified motifs. Unaligned sequences of 29 Centroid sequences from Saccharomycetaceae species were analyzed using MEME [62] to identify amino acid sequence motifs that occur more often than expected by chance. The top 3 most statistically significant motifs are shown. Motif 1 is present in almost every sequence, whereas Motifs 2 and 3 are largely restricted to non-WGD and post-WGD species, respectively. To avoid an over-representation of Saccharomyces sequences, only S. cerevisiae Ynl018c was included from this genus. (PDF) [file pgen.1010525.s007.pdf]

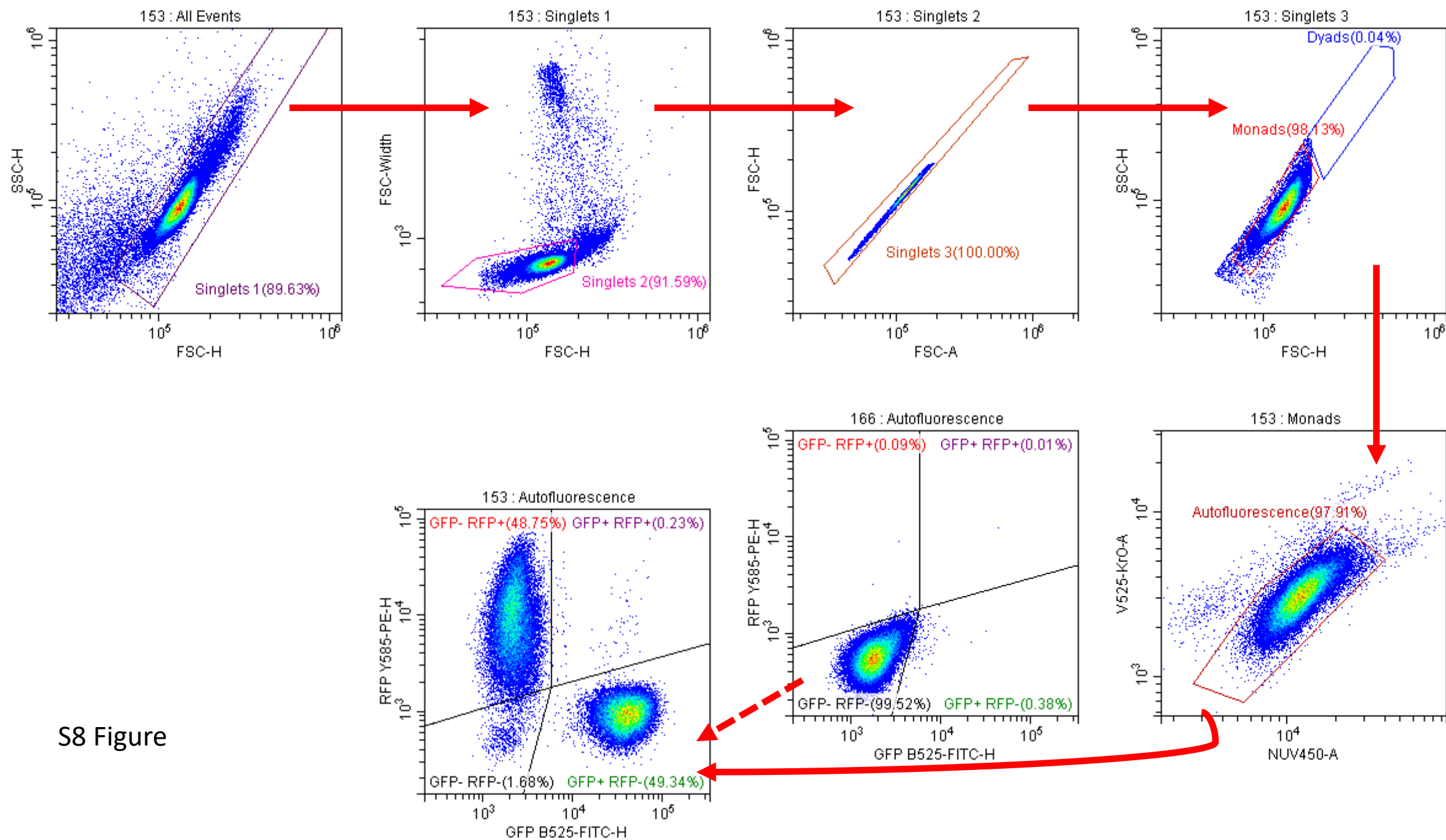

S8 Figure

Supplement: S8 Fig — An initial scatter gate was used to remove debris and noise (FSC-H vs SSC-H). Single events (vs. aggregates) were then selected using a combination of gates (FSC-H vs FSC-Width and FSC-A vs FSC-H). The singlets were then separated into monads and dyads/tetrads (FSC-H vs SSC-H). The monad population was then gated for auto-fluorescence “noise” (NUV450-A vs V525-KrO-A). The gates for GFP (B525-FITC-H) and RFP (Y585-PE-H) expression were then set based on the expression profile of the negative control population (strain LS166). Percent expression in each quadrant was then scored. This example shows strain LS153, with 49.34% of monads scored as expressing only PDIT1-GFP (GFP+ RFP-) and 48.75% scored as expressing only PDIT1-RFP (GFP- RFP+). (PDF) [file pgen.1010525.s008.pdf]
