# Supplementary material for: A widespread inversion polymorphism conserved among Saccharomyces species is caused by recurrent homogenization of a sporulation gene family
Source: PLoS Genet. 2022 Nov 28;18(11):e1010525. doi: 10.1371/journal.pgen.1010525 (PMC9731477; doi:10.1371/journal.pgen.1010525)
Supplement: S5 Fig — Green tracks indicate rightward transcription, and red tracks indicate leftward transcription. (A) Meiosis and sporulation time course from Brar et al. [55]. Their study combined two time courses (a “traditional meiosis time course” with points A-V, and a time course after artificial induction of the late meiosis regulator NDT80, with points 1–18), into a single master time course with points in the order shown. To save space, data from every second time point is shown. Data from NCBI SRA accession numbers SRR387838 to SRR387870. (B) Meiosis time course data from Gould et al. [56] after induction of NDT80. In strain SK1, IRL is interrupted by a Ty3 element [51]. Data from NCBI SRA accession numbers SRR2831307 to SRR2831321. (C) Transcription during vegetative exponential growth of S288C wildtype haploid cells. Data from Wery et al. [57], NCBI SRA accession number SRR2045245. (PDF) [file pgen.1010525.s005.pdf]

**(A)** Brar et al. (2012). Master timecourse of meiosis-sporulation.

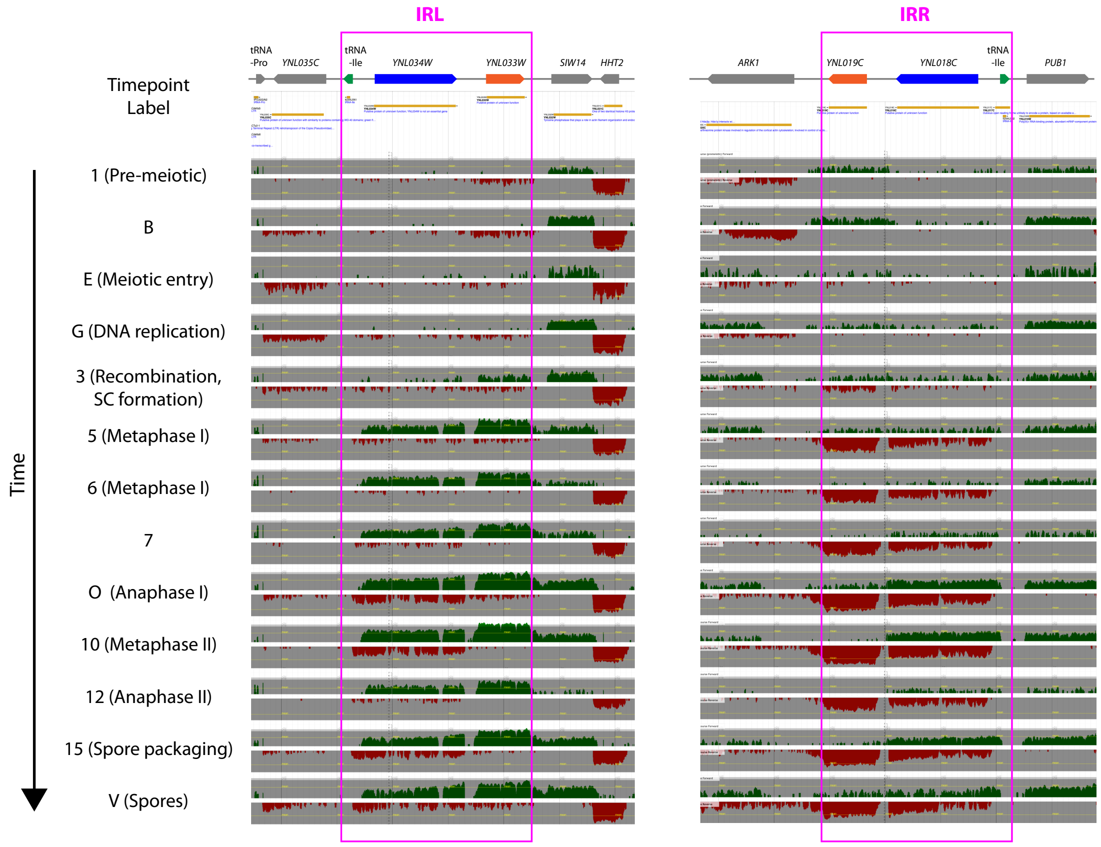


**(B)** Gould et al. (2016). Meiosis timecourse.

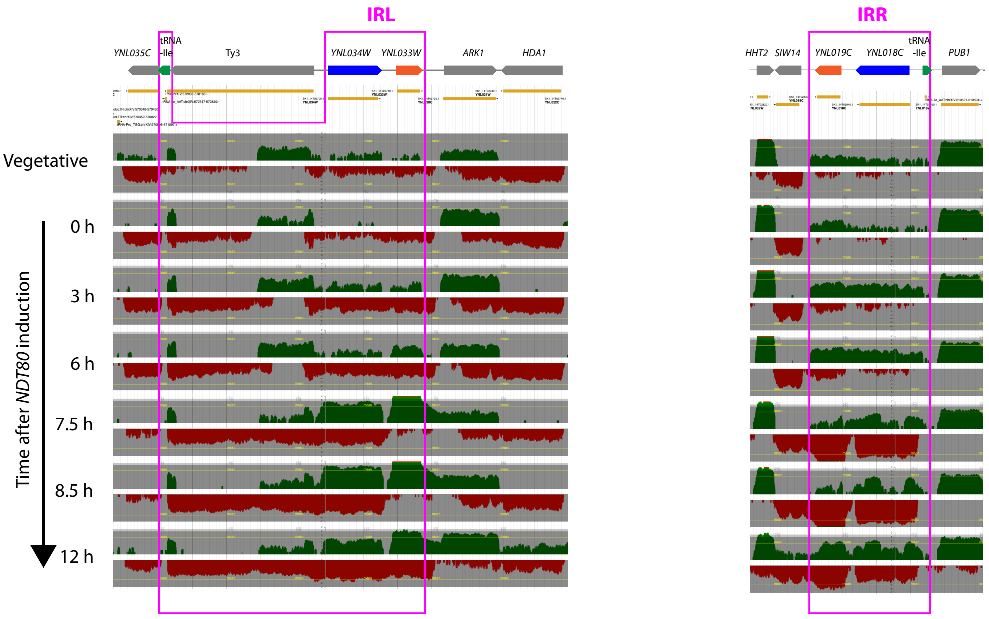


**(C)** Wery et al. (2016). Vegetative growth (haploid cells).

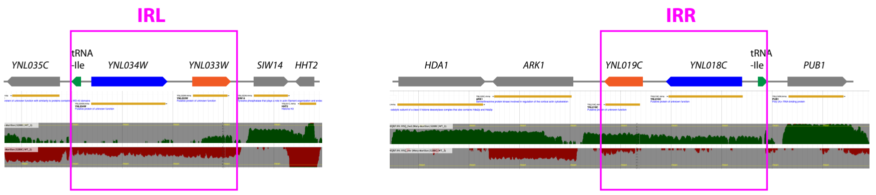


S5 Figure
