# Supplementary material for: A widespread inversion polymorphism conserved among Saccharomyces species is caused by recurrent homogenization of a sporulation gene family
Source: PLoS Genet. 2022 Nov 28;18(11):e1010525. doi: 10.1371/journal.pgen.1010525 (PMC9731477; doi:10.1371/journal.pgen.1010525)
Supplement: S6 Fig — The tree was inferred as described in Materials and Methods. Circled numbers indicate pairs or trios of genes that have orthologous (syntenic) genomic locations [61]. (PDF) [file pgen.1010525.s006.pdf]

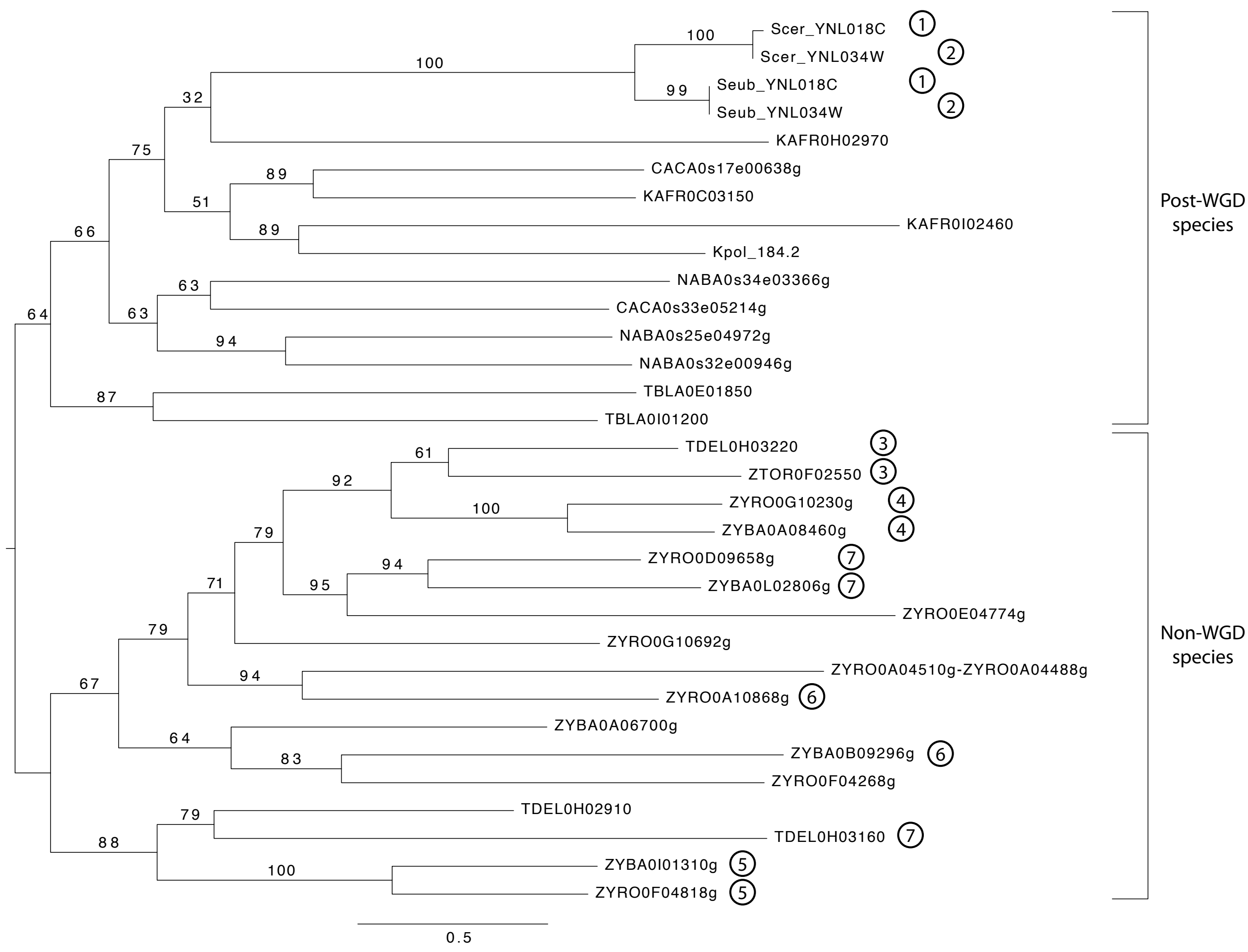

Scer, *Saccharomyces cerevisiae*

Seub, *Saccharomyces eubayanus*

NABA, *Nakaseomyces bacillisporus*

KAFR, *Kazachstania africana*

CACA, *Candida castellii*

TBLA, *Tetrapisispora blattae*

Kpol, *Vanderwaltozyma polyspora*

ZYRO, *Zygosaccharomyces rouxii*

ZYBA, *Zygosaccharomyces bailii*

TDEL, *Torulaspora delbrueckii*

ZTOR, *Zygotorulaspora mrakii*

S6 Figure
