# Supplementary material for: A widespread inversion polymorphism conserved among Saccharomyces species is caused by recurrent homogenization of a sporulation gene family
Source: PLoS Genet. 2022 Nov 28;18(11):e1010525. doi: 10.1371/journal.pgen.1010525 (PMC9731477; doi:10.1371/journal.pgen.1010525)
Supplement: S2 Table — Sequence data are from Lee et al. [23]. (PDF) [file pgen.1010525.s010.pdf]

S2 Table. FF region orientations in 24 natural isolates of *S. cerevisiae* from Taiwan. Sequence data are from Lee et al. (2022), NCBI Bioproject PRJNA755173.

Yeast isolate ID	Source ID	FF region orientation	Location Description	Ploidy	Clonal group	SRA accession number	Example of informative read
LY0215	LY0215	INV	Taiwan, Taichung, Heping: Wuling farm	2	TW4-(A)	SRR18726966	SRR18726966.160955
PD15A	SP00001	INV	Taiwan, Taipei, Daan: National Taiwan University campus	2	TW5-(A)	SRR18726965	SRR18726965.4605
PD21B	SP00004	INV	Taiwan, Taipei, Daan: National Taiwan University campus	2	TW5-(B)	SRR18726954	SRR18726954.53170
PD41A	SP00016	INV	Taiwan, Island, Dongsha: Da-Wang temple	2	TW_Mosaic-(F)	SRR18726963	SRR18726963.78401
PD27A	SP00101	REF	Taiwan, Hualien, Fuli: Cilamitay	2	TW_Mosaic-(A)	SRR18726949	SRR18726949.9683
PD28A	SP00104	REF	Taiwan, Taitung, Beinan: Castanopsis carlesii seed orchard	2	TW_Mosaic-(B)	SRR18726948	SRR18726948.30688
PD31A	SP00127	INV	Taiwan, Taitung, Dawu: Sanzhuku Recreation Farm	2	TW2a-(A)	SRR18726947	SRR18726947.44153
PD33A	SP00134	INV	Taiwan, Taitung, Daren: Shouka	2	TW2a-(A)	SRR18726946	SRR18726946.31862
PD35A	SP00147	INV	Taiwan, Nantou, Puli: Shikeng Road	2	TW2a-(B)	SRR18726945	SRR18726945.53769
PD36A	SP00147	REF	Taiwan, Nantou, Puli: Shikeng Road	3	TW4-(B)	SRR18726944	SRR18726944.50986
PD38A	SP00147	REF	Taiwan, Nantou, Puli: Shikeng Road	2	TW_Mosaic-(D)	SRR18726943	SRR18726943.123910
PD40A	SP00153	INV	Taiwan, Nantou, Puli: National Chi Nan University	2	TW3-(E)	SRR18726964	SRR18726964.25486
RG06	SP00171	INV	Taiwan, Pingtung, Majia: Litingshan	2	TW3-(A)	SRR18726962	SRR18726962.37791
SR18	SP00314	INV	Taiwan, Yilan, Yuanshan: Fushan Botanical Garden	2	TW2a-(C)	SRR18726951	SRR18726951.62793
S5	SPA0048	INV	Taiwan, Taipei, Nangang: Nangangshan Hiking Trail	2	TW2b	SRR18726957	SRR18726957.40551
S7	SPA0050	INV	Taiwan, Taipei, Nangang: Nangangshan Hiking Trail	2	CHN-VI/VII.1-(B)	SRR18726956	SRR18726956.87019
S8	SPA0080	INV	Taiwan, Yilan, Yuanshan: Fushan Botanical Garden	2	TW_Mosaic-(G)	SRR18726955	SRR18726955.1298
S10	SPA0084	INV	Taiwan, Yilan, Yuanshan: Fushan Botanical Garden	2	CHN-VI/VII.1-(A)	SRR18726961	SRR18726961.43614
S13	SPA0107	INV	Taiwan, Yilan, Yuanshan: Fushan Botanical Garden	2	TW_Mosaic-(I)	SRR18726960	SRR18726960.16255
S14	SPA0108	INV	Taiwan, Yilan, Yuanshan: Fushan Botanical Garden	2	CHN-VIII-(A)	SRR18726959	SRR18726959.99788
S16	SPA0138	REF	Taiwan, Taipei, Nangang: Academia Sinica	2	CHN-VIII-(B)	SRR18726958	SRR18726958.52270
XI06	SPA0305	INV	Taiwan, Yilan, Datong: Tuling	2	TW2a-(D)	SRR18726950	SRR18726950.97072
SPA0342	SPA0342	REF	Taiwan, Yilan, Datong: Tuling	2	TW1-(A)	SRR18726953	SRR18726953.13485
SPA0344	SPA0344	REF	Taiwan, Yilan, Datong: Tuling	2	TW1-(A)	SRR18726952	SRR18726952.51669

Total: 7 REF isolates (29%), 17 INV isolates (71%).

For further details about yeast isolates and sample locations, see Table S6 of Lee et al. (2022). FF region orientation was determined by TBLASTN searches against unassembled long read (Oxford Nanopore) reads downloaded from the NCBI Sequence Reads Archive (SRA), using *YNL035C, SIW14, ARK1* and *PUB1* protein sequences as queries. An example of a sequence read that is informative about the orientation of the FF region is listed for each isolate. No REF/INV heterozygotes were detected.
