# Supplementary material for: A widespread inversion polymorphism conserved among Saccharomyces species is caused by recurrent homogenization of a sporulation gene family
Source: PLoS Genet. 2022 Nov 28;18(11):e1010525. doi: 10.1371/journal.pgen.1010525 (PMC9731477; doi:10.1371/journal.pgen.1010525)
Supplement: S3 Table — (PDF) [file pgen.1010525.s011.pdf]

S3 Table. PCR primer sequences for FF region amplification.

**Primer**	**Sequence**	**Nearest gene**
ScUf	CCCGAAAGAATTCGATTCAA	*YNL035C*
ScDr	CAAGCCTCTTCGTTCACCTC	*PUB1*
ScUf2	ACAGTAAGACGAGCCAGTGC	*YNL035C*
ScUr2	CGTCCTGCCATACAAATGCG	*HHT2*
ScDf2	GCATGCAACGCTTGAATTAG	*ARK1*
ScDr2	CGCAACGCGTAAACCTTATG	*PUB1*
SuUf	TCAGCCCTGTTAGAAGTCCA	*YNL035C*
SuUr	GTTTGATATGAACCCATTTCCC	*SIW14*
SuDf	CAACTTCATAGCATCTACTTCCG	*ARK1*
SuDr	GTCCAGATTACAAAGCGAGG	*PUB1*
