# Supplementary material for: A widespread inversion polymorphism conserved among Saccharomyces species is caused by recurrent homogenization of a sporulation gene family
Source: PLoS Genet. 2022 Nov 28;18(11):e1010525. doi: 10.1371/journal.pgen.1010525 (PMC9731477; doi:10.1371/journal.pgen.1010525)
Supplement: S4 Table — (PDF) [file pgen.1010525.s012.pdf]

**S4 Table.** Genetically modified *S. cerevisiae* strains used in this study.

| Strain Name | Genotype | Reference |
|---|---|---|
| **Haploid strains** | | |
| YDP1285 | Y55 MATa ura3::KANMX | Rogers et al. |
| YDP1307 | Y55 MATα leu2::HYGMX | Rogers et al. |
| YDP1343 | Y55 MATα leu2::HYGMX dit1::RFP_LEU2 | Rogers et al. |
| YDP1351 | Y55 MATa ura3::KANMX dit1::GFP_URA3 | Rogers et al. |
| YDP1399 | Y55 MATα leu2::HYGMX ynl018c:: $P_{DIT1}$-GFP_LEU2 | Rogers et al. |
| YDP1412 | Y55 MATa ura3::KANMX ynl034w::$P_{DIT1}$-GFP_URA3 | Rogers et al. |
| LS022 | Y55 MATa ura3::KANMX ynl011c::$P_{DIT1}$-GFP_URA3 | This work |
| LS023 | Y55 MATα leu2::HYGMX ynl011c:: $P_{DIT1}$-GFP_LEU2 | This work |
| LS071 | Y55 MATa ura3::KANMX ynl011c::$P_{DIT1}$-GFP_URA3 ynl018cΔ ynl034wΔ | This work |
| LS072 | Y55 MATa ura3::KANMX ynl011c::$P_{DIT1}$-GFP_URA3 ynl019cΔ ynl033wΔ | This work |
| LS142 | Y55 MATα leu2::KANMX | This work |
| LS143 | Y55 MATα leu2::KANMX ynl018c:: $P_{DIT1}$-GFP_LEU2 | This work |
| LS144 | Y55 MATα leu2::KANMX ynl011c:: $P_{DIT1}$-GFP_LEU2 | This work |
| LS148 | Y55 MATa ura3::KANMX ynl011c::$P_{DIT1}$-GFP_URA3 tRNA-ile_ynl018c_ynl019cΔ tRNA-ile_ynl034w_ynl033wΔ | This work |
| LS149 | Y55 MATα leu2::KANMX ynl011c:: $P_{DIT1}$-GFP_LEU2 tRNA-ile_ynl018c_ynl019cΔ tRNA-ile_ynl034w_ynl033wΔ | This work |
| LS162 | Y55 MATα leu2::KANMX ynl011c:: $P_{DIT1}$-GFP_LEU2 ynl018cΔ ynl034wΔ | This work |
| LS163 | Y55 MATα leu2::KANMX ynl011c:: $P_{DIT1}$-GFP_LEU2 ynl019cΔ ynl033wΔ | This work |
| LS178 | Y55 MATa ura3::KANMX ynl011c::$P_{DIT1}$-GFP_URA3 tRNA-ile_ynl018c_ynl019cΔ::YNL018C_YNL019C tRNA-ile_ynl034w_ynl033wΔ | This work |
| LS179 | Y55 MATα leu2::KANMX ynl011c:: $P_{DIT1}$-GFP_LEU2 tRNA-ile_ynl018c_ynl019cΔ::YNL018C tRNA-ile_ynl034w_ynl033wΔ | This work |
| LS181 | Y55 MATa ura3::KANMX ynl011c::$P_{DIT1}$-GFP_URA3 tRNA-ile_ynl018c_ynl019cΔ tRNA-ile_ynl034w_ynl033wΔ ho::YNL018C_YNL019C | This work |
| LS189 | Y55 MATa ura3::KANMX ynl011c::$P_{DIT1}$-GFP_URA3 tRNA-ile_ynl018c_ynl019cΔ tRNA-ile_ynl034w_ynl033wΔ ho::YNL018C | This work |
| LS190 | Y55 MATα leu2::KANMX ynl011c:: $P_{DIT1}$-GFP_LEU2 tRNA-ile_ynl018c_ynl019cΔ tRNA-ile_ynl034w_ynl033wΔ ho::YNL018C | This work |
| LS196 | Y55 MATα leu2::KANMX ynl011c:: $P_{DIT1}$-GFP_LEU2 tRNA-ile_ynl018c_ynl019cΔ::*S. paradoxus* YNL018C tRNA-ile_ynl034w_ynl033wΔ | This work |
| LS199 | Y55 MATα leu2::KANMX ynl011c:: $P_{DIT1}$-GFP_LEU2 tRNA-ile_ynl018c_ynl019cΔ::*S. paradoxus* YNL018C_YNL019C tRNA-ile_ynl034w_ynl033wΔ | This work |
| LS201 | Y55 MATα leu2::KANMX ynl011c:: $P_{DIT1}$-GFP_LEU2 tRNA-ile_ynl018c_ynl019cΔ tRNA-ile_ynl034w_ynl033wΔ ho::*S. paradoxus* YNL018C_YNL019C | This work |
| **Diploid strains** | | |
| LS150 | LS148 × LS149 | This work |
| LS151 | LS022 × LS149 | This work |
| LS152 | LS148 × LS144 | This work |
| LS153 | LS022 × LS144 | This work |
| LS154 | YDP1412 × LS149 | This work |
| LS166 | YDP1285 × LS142 | This work |
| LS168 | LS071 × LS162 | This work |
| LS169 | LS072 × LS163 | This work |
| LS170 | LS148 × LS143 | This work |
| LS183 | LS148 × LS179 | This work |
| LS185 | LS178 × LS149 | This work |
| LS187 | LS181 × LS149 | This work |
| LS191 | LS148 × LS190 | This work |
| LS192 | LS189 × LS149 | This work |
| LS197 | LS148 × LS196 | This work |
| LS202 | LS148 × LS199 | This work |
| LS203 | LS148 × LS201 | This work |
| LS206 | LS022 × LS196 | This work |
| LS207 | LS022 × LS199 | This work |
| LS209 | YDP1412 × LS199 | This work |
