# Supplementary material for: A widespread inversion polymorphism conserved among Saccharomyces species is caused by recurrent homogenization of a sporulation gene family
Source: PLoS Genet. 2022 Nov 28;18(11):e1010525. doi: 10.1371/journal.pgen.1010525 (PMC9731477; doi:10.1371/journal.pgen.1010525)
Supplement: S5 Table — (PDF) [file pgen.1010525.s013.pdf]

S5 Table. Oligonucleotides used for strain construction.

#	Name	Sequence (5' → 3')
5	pJBo9 guide rev	atacgactcactatagggcg
51	YNL011C fwd	GGGTCCACAAGTGAAATTCTACGAATAGTTGGTGGCCCAGcgtttcggtgatgacggtgaaaa
52	YNL011C rev	CAATAGATGCGTCTAGAGAACCTAAAAACAATCTAGCACCaggtgggacaatgattgcaaagg
55	YNL018C/34W guide fwd	gcagtgaaagataggtgaccAGGTTGGGCTAGACTTTCAAgttttagaaagcgaaagctt
60	YNL019C/33W guide fwd	gcagtgaaagataggtgaccTGCTTTACTCCAGAGAATCAgttttagaaagcgaaagctt
94	YNL018C/34W del rTOP	GTTGAAAAAATTTGCTAAAGCGTTGGTCTAATTTTATTTTTGCCCACATAACCGTCAGAA
95	YNL018C/34W del rBOT	GGTTTGTTCAGCCGTGCCAAACAGTTCGAACTATCAGAACTTCTGACGGTTATGTGGGCA
96	YNL019C/33W del rTOP	AAACACAAGTTGATACAACGAGGCTGTCAATAGCAAGAGCGTGCGCGAGAATGGAGCTGC
97	YNL019C/33W del rBOT	CATGTAGGGGTAAGTTGGTGTATGGTGTGTACAGCCAGGAGCAGCTCCATTCTCGCGCAC
99	YNL018 com guide fwd	gcagtgaaagataggtgaccAGCATAAGGTTTACGCGTTGgttttagaaagcgaaagctt
113	leu2::kanmx fwd	GCTATTTGGATATTTTATATTGACTTTCGTGTACATTGATaattaaggcgcgccagatc
114	leu2::kanmx rev	CGTCTACCCTATGAACATATTCCATTTTGTAATTTCGTGTatcgatgaattcgagctcg
120	18_19 IR rTOP	aagcctcttcgttcacctcccttgtaacggcagtgggaagggtaagcaaaatatcgactt
121	18_19 IR rBOT	gcttttttagacattaatttttcccttatgtagctggcaaagtcgatattttgcttacc
128	34_33 IR rTOP	gcactgtagtcccatctaacatttttacaatctggactaagaaaactttttttcaaatgg
129	34_33 IR rBOT	tcgtgtttctctcttcaagttaatactatggagaccttggccatttgaaaaaaagttttc
167	18_19 comp RT fwd	cgttaggataattaatcttcagacgtttagtaagaacattgtgcttacaaattgttatctgttttccaccctccctcc
169	18 comp RT rev2	gcttttttagacattaatttttcccttatgtagctggcaaagtcgatatagtctcgcgcacgctcttgctattg
171	18_19 comp RT rev2	gcttttttagacattaatttttcccttatgtagctggc
172	HO comp guide fwd	gcagtgaaagataggtgaccTGGCTTGGATGCTTGGTCTGgttttagaaagcgaaagctt
174	18_19 HO comp RT fwd	TGTTTTATCTAAATTTCTCACTGGACGTAGTGACCTTGTAACTCCTGCTGattgttatctgttttccaccctccctcc
175	18 HO comp RT rev	GGATCCAAGCTATCTACTGAGATTTCTGGCTCTTTTGTTGTACCGTCACCagtctcgcgcacgctcttgctattg
176	18_19 HO comp RT rev	GGATCCAAGCTATCTACTGAGATTTCTGGCTCTTTTGTTGTACCGTCACCccagaagagagtagataaaaagccg
181	Spx 18_19 locus F	cgttaggataattaatcttcagacgtttagtaagaacattgtgcttacaaaaggttttctgtttttcacttccacttcac
182	Spx 18 locus R	gcttttttagacattaatttttcccttatgtagctggcaaagtcgatatattctcgcgtatgctcttgctattgg
183	Spx 18_19 locus R	gcttttttagacattaatttttcccttatgtagctggcaaagtcgatatcctttcaactcaattatatgttgaccttgc
184	Spx 18_19 HO F	TGTTTTATCTAAATTTCTCACTGGACGTAGTGACCTTGTAACTCCTGCTGaaggttttctgtttttcacttccacttcac
186	Spx 18_19 HO R	GGATCCAAGCTATCTACTGAGATTTCTGGCTCTTTTGTTGTACCGTCACCcctttcaactcaattatatgttgaccttgc
