# Supplementary material for: A widespread inversion polymorphism conserved among Saccharomyces species is caused by recurrent homogenization of a sporulation gene family
Source: PLoS Genet. 2022 Nov 28;18(11):e1010525. doi: 10.1371/journal.pgen.1010525 (PMC9731477; doi:10.1371/journal.pgen.1010525)
Supplement: S2 Fig — Genes in the FF region on S. cerevisiae chromosome XIV are named in red. Dots represent protein-coding genes, and triangles represent tRNA genes with orientations as shown. Homologous genes (orthologs, or pairs of paralogs in post-WGD species) are drawn in the same row. The three “Non-WGD species” columns in the center show the order of genes in two species that did not undergo WGD (Lachancea kluyveri and Zygotorulaspora mrakii), and in the inferred last common ancestor of the WGD clade [97]. This gene order is compared to two post-WGD species: S. cerevisiae and Candida castellii [98]. Each post-WGD species has two paralogous chromosomal regions (Track A and Track B), corresponding to parts of chromosomes XIV and IX/IV in S. cerevisiae, and parts of scaffolds s05 and s21/s27 in C. castellii. The FF region of S. cerevisiae is drawn in INV orientation, and its gene order shows similarities with the non-WGD species, with C. castellii Track A, and with Track B from both of the post-WGD species. YNL018C/YNL034W and YNL019C/YNL033W do not have orthologs in any of the other species shown, but a pair of tRNA-Ile genes in opposite orientations forms a small IR in the non-WGD species and in S. cerevisiae Track B (chromosome IX), as shown by the gray shading. The species shown were chosen because they have relatively little rearrangement in this area of the genome. One inversion shared by S. cerevisiae and C. castellii, and one inversion unique to S. cerevisiae, are visible. A few species-specific genes have been omitted for clarity, and the noncoding RNA genes NME1 and SNR66 are not shown because they are not annotated in all genomes. (PDF) [file pgen.1010525.s002.pdf]

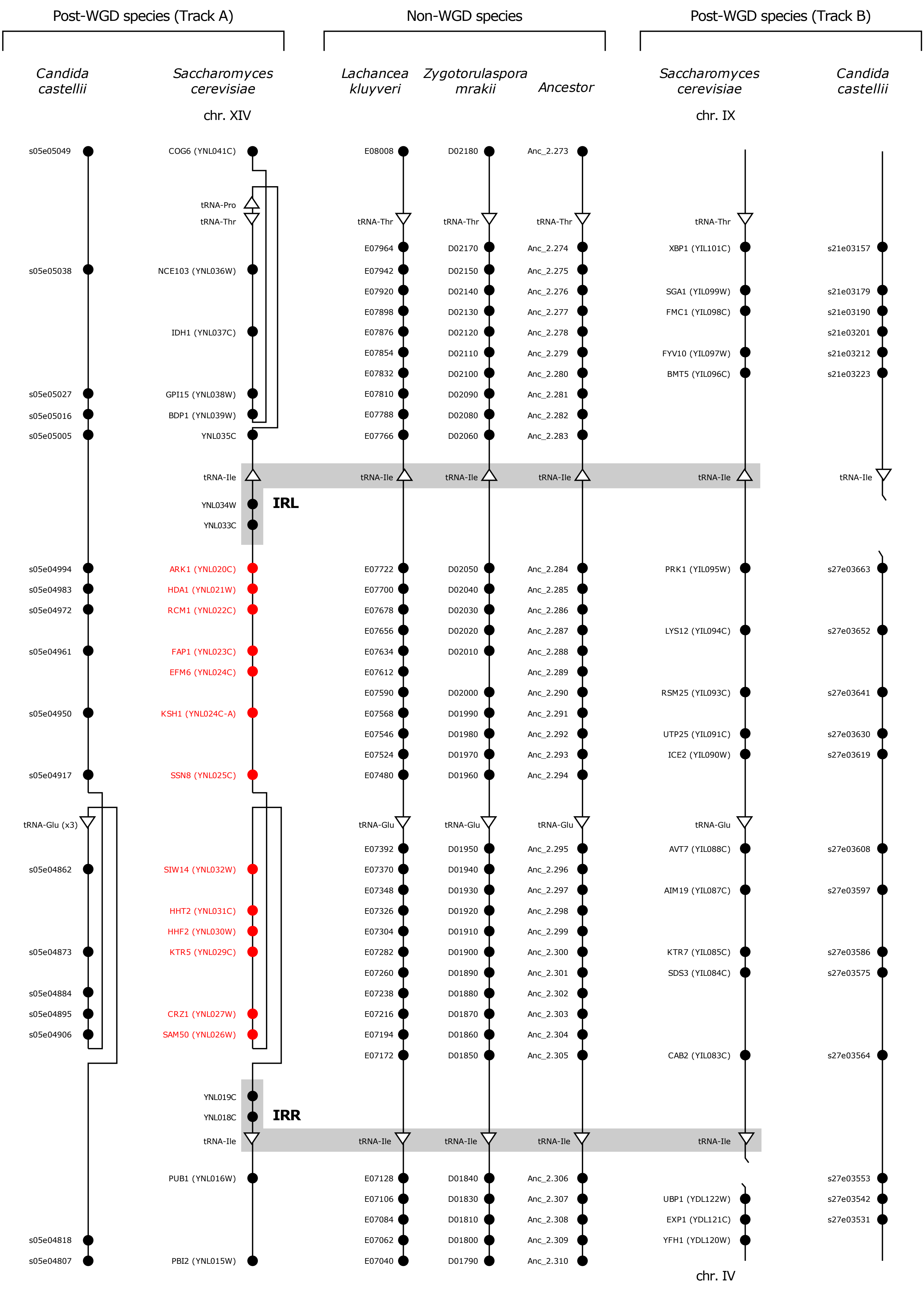
Post-WGD species (Track A)
Non-WGD species
Post-WGD species (Track B)
Candida castellii
Saccharomyces cerevisiae
chr. XIV
Lachancea kluyveri
Zygotorulaspora mrakii
Ancestor
Saccharomyces cerevisiae
chr. IX
Candida castellii
s05e05049
s05e05038
s05e05027
s05e05016
s05e05005
s05e04994
s05e04983
s05e04972
s05e04961
s05e04950
s05e04917
tRNA-Glu (x3)
s05e04862
s05e04873
s05e04884
s05e04895
s05e04906
s05e04818
s05e04807
COG6 (YNL041C)
tRNA-Pro
tRNA-Thr
NCE103 (YNL036W)
IDH1 (YNL037C)
GPI15 (YNL038W)
BDP1 (YNL039W)
YNL035C
tRNA-Ile
YNL034W
YNL033C
IRL
ARK1 (YNL020C)
HDA1 (YNL021W)
RCM1 (YNL022C)
FAP1 (YNL023C)
EFM6 (YNL024C)
KSH1 (YNL024C-A)
SSN8 (YNL025C)
SIW14 (YNL032W)
HHT2 (YNL031C)
HHF2 (YNL030W)
KTR5 (YNL029C)
CRZ1 (YNL027W)
SAM50 (YNL026W)
YNL019C
YNL018C
IRR
tRNA-Ile
PUB1 (YNL016W)
PBI2 (YNL015W)
E08008
tRNA-Thr
E07964
E07942
E07920
E07898
E07876
E07854
E07832
E07810
E07788
E07766
tRNA-Ile
E07722
E07700
E07678
E07656
E07634
E07612
E07590
E07568
E07546
E07524
E07480
tRNA-Glu
E07392
E07370
E07348
E07326
E07304
E07282
E07260
E07238
E07216
E07194
E07172
tRNA-Ile
E07128
E07106
E07084
E07062
E07040
D02180
tRNA-Thr
D02170
D02150
D02140
D02130
D02120
D02110
D02100
D02090
D02080
D02060
tRNA-Ile
D02050
D02040
D02030
D02020
D02010
D02000
D01990
D01980
D01970
D01960
tRNA-Glu
D01950
D01940
D01930
D01920
D01910
D01900
D01890
D01880
D01870
D01860
D01850
tRNA-Ile
D01840
D01830
D01810
D01800
D01790
Anc_2.273
tRNA-Thr
Anc_2.274
Anc_2.275
Anc_2.276
Anc_2.277
Anc_2.278
Anc_2.279
Anc_2.280
Anc_2.281
Anc_2.282
Anc_2.283
tRNA-Ile
Anc_2.284
Anc_2.285
Anc_2.286
Anc_2.287
Anc_2.288
Anc_2.289
Anc_2.290
Anc_2.291
Anc_2.292
Anc_2.293
Anc_2.294
tRNA-Glu
Anc_2.295
Anc_2.296
Anc_2.297
Anc_2.298
Anc_2.299
Anc_2.300
Anc_2.301
Anc_2.302
Anc_2.303
Anc_2.304
Anc_2.305
tRNA-Ile
Anc_2.306
Anc_2.307
Anc_2.308
Anc_2.309
Anc_2.310
tRNA-Thr
XBP1 (YIL101C)
SGA1 (YIL099W)
FMC1 (YIL098C)
FYV10 (YIL097W)
BMT5 (YIL096C)
tRNA-Ile
PRK1 (YIL095W)
LYS12 (YIL094C)
RSM25 (YIL093C)
UTP25 (YIL091C)
ICE2 (YIL090W)
tRNA-Glu
AVT7 (YIL088C)
AIM19 (YIL087C)
KTR7 (YIL085C)
SDS3 (YIL084C)
CAB2 (YIL083C)
tRNA-Ile
UBP1 (YDL122W)
EXP1 (YDL121C)
YFH1 (YDL120W)
chr. IV
s21e03157
s21e03179
s21e03190
s21e03201
s21e03212
s21e03223
tRNA-Ile
s27e03663
s27e03652
s27e03641
s27e03630
s27e03619
s27e03608
s27e03597
s27e03586
s27e03575
s27e03564
s27e03553
s27e03542
s27e03531

S2 Figure
